# Efficacy of the additional use of subgingival air-polishing with erythritol powder in the treatment of periodontitis patients: a randomized controlled clinical trial. Part II: effect on sub-gingival microbiome

**DOI:** 10.1007/s00784-022-04811-4

**Published:** 2022-12-20

**Authors:** Magda Mensi, Elisabetta Caselli, Maria D’Accolti, Irene Soffritti, Roberto Farina, Eleonora Scotti, Maria Elena Guarnelli, Chiara Fabbri, Gianluca Garzetti, Silvia Marchetti, Annamaria Sordillo, Leonardo Trombelli

**Affiliations:** 1 Section of Periodontics, School of Dentistry, Department of Surgical Specialties, Radiological Science and Public Health, University of Brescia, P.Le Spedali Civili 1, 25123 Brescia, Italy; 2 U.O.C. Odontostomatologia - ASST Degli Spedali Civili Di Brescia, Brescia, Italy; 3 Section of Microbiology and LTTA, Department of Chemical, Pharmaceutical and Agricultural Sciences, University of Ferrara, Ferrara, Italy; 4 Research Centre for the Study of Periodontal and Peri-Implant Diseases, University of Ferrara, Ferrara, Italy; 5 Operative Unit of Dentistry, Azienda Unità Sanitaria Locale (AUSL), Ferrara, Italy

**Keywords:** Periodontal disease, Non-surgical therapy, Air-polishing, Microbiota

## Abstract

**Objectives:**

To date, scarce evidence exists around the application of subgingival air-polishing during treatment of severe periodontitis. The aim of this study was to evaluate the effect on the health-related and periodontitis-related subgingival microbiome of air-polishing during non-surgical treatment of deep bleeding pockets in stage III–IV periodontitis patients.

**Materials and methods:**

Forty patients with stage III–IV periodontitis were selected, and pockets with probing depth (PD) 5–9 mm and bleeding on probing were selected as experimental sites. All patients underwent a full-mouth session of erythritol powder supragingival air-polishing and ultrasonic instrumentation. Test group received additional subgingival air-polishing at experimental sites. Subgingival microbial samples were taken from the maxillary experimental site showing the deepest PD at baseline. Primary outcome of the first part of the present study was the 3-month change in the number of experimental sites. Additional analysis of periodontal pathogens and other sub-gingival plaque bacteria sampled at one experimental site at baseline and 3 months following treatment was performed through a real-time quantitative PCR microarray.

**Results:**

In the test group, a statistical increase of some health-related species was observed (*Abiotropha defectiva, Capnocytophaga sputigena, and Lautropia mirabilis*), together with the decrease of pathogens such as of *Actinomyces israelii*, *Catonella morbi*, *Filifactor alocis*, *Porphyromonas endodontalis*, *Sele-nomonas sputigena*, *Tannerella forsythia*, *Treponema denticola*, and *Treponema socranskii*. In the control group, statistical significance was found only in the decrease of *Filifactor alocis*, *Tannerella forsythia*, and *Treponema socranskii*.

**Conclusions:**

The addition of erythritol-chlorhexidine powder seems to cause a shift of the periodontal micro-biome toward a more eubiotic condition compared to a conventional treatment. The study was registered on Clinical Trials.gov (NCT04264624).

**Clinical relevance:**

Subgingival air-polishing could help re-establishing a eubiotic microbioma in deep bleeding periodontal pockets after initial non-surgical treatment.

**Supplementary Information:**

The online version contains supplementary material available at 10.1007/s00784-022-04811-4.

## Introduction

The mouth supports the second largest and most diverse microbial community found in the body after the gut, including approximately 700 bacterial species. According to the expanded Human Oral Microbiome Database (HOMD), only 57% of oral bacteria have been officially named, whereas 13% are yet unnamed although cultivated, and 30% are uncultivated[1]. For this reason, the current trend in oral microbiome studies is greatly taking advantage of culture-independent technologies, including PCR microarrays, next-generation sequencing, and whole-genome sequencing, metabolomics, and preoteomics, which can overcome the limitations of culture-dependent protocols and provide species identification thanks to the continuous expansions of databases of microbial genetic sequences[2, 3]. The unique microbiomes of the saliva, tongue, buccal mucosa, teeth surfaces, gums, palate, and both subgingival and supragingival plaque have all been characterized, showing high diversity between diverse niches, despite a common healthy oral microbiome can be recognized in young healthy population[4]. Around 500 different bacterial species can be encountered in the subgingival flora[4, 5]. Of major importance is the interaction between this microbiome, the host, and environmental factors[6]. Maternal transmission, genetics, and environmental factors such as diet, oral hygiene, smoking, medications, and stress can influence the composition and function of the oral microbiome[3]. The oral ecosystem is constantly challenged and, while a certain natural resilience is observed, when a perturbation passes a certain threshold (e.g., plaque accumulation, reduced salivary flow, immunodeficiency), a shift takes place toward oral disease, with a change in composition and increase in complexity[7, 8]. This shift, together with the interaction with the host's inflammatory response[9], seems to be the determinant for destruction/re-organization of the periodontal tissues[10].

Periodontal disease has been traditionally linked to a higher subgingival bacterial count and to a prominent relative presence of certain pathogenic bacteria, such as *P. intermedia*, *P. gingivalis*, *T*. *Forsythia*, and *T. denticola*, members of the so-called orange and red complex[5], and Gram-negative bacteria such as A. *actinomycetemcomitans*[11, 12]. More recently, new pathogens have been identified and associated with periodontal disease, such as *Selenomonas*, *Synergistes*, *Desulfobulbus*, and *F*. *alocis*[13]. Recent studies have further confirmed significant differences in microbiomes of periodontitis subjects compared to healthy controls, indicating *Treponema*, *TG5*, *Desulfobulbus*, *Catonella*, *Bacteroides*, *Aggregatibacter*, *Peptostreptococcus*, and *Eikenella* as periodontitis biomarkers, while *Veillonella*, *Corynebacterium*, *Neisseria*, *Rothia*, *Paludibacter*, *Capnocytophaga*, and *Kingella* as signatures of healthy periodontium[3, 14, 15]. In addition, recent studies highlighted that the periodontal and peri-implant dysbiosis correlates with alterations in the bacterial relationships between pathobionts and healthy microbiota, suggesting that the community structure and possible negative correlations should be given more concern in future approaches[16].

Non-surgical periodontal therapy is proven to induce a positive shift in the microflora through the mechanical removal of biofilm and a trigger of the immunological response[17, 18]. Effective mechanical disruption of biofilm is fundamental as bacteria as protected by a matrix with complex viscoelastic properties: if not enough energy is applied, the biofilm is expanded but not removed[19]. In recent years, technological innovation has allowed the introduction of new means of treating periodontal disease, with a focus on minimal invasiveness and maximum preservation of periodontal tissues, while maintaining the efficacy. From traditional scaling and root planing, we are now shifting toward the more conservative root surface debridement[20]. Moreover, different adjunctive therapies have been introduced and studied with the aim of improving the clinical and microbiological outcomes of non-surgical therapy, among them sub-gingival air-polishing with different types of powders, from sodium bicarbonate to the most recent erythritol[21].

To date, a few studies have investigated the clinical effects of air-polishing in the initial treatment of patients with periodontitis[21–25], but only one analyzed its microbiological effects, finding that air-polishing induced a more marked reduction of *P. gingivalis*[24]. Moreover, there is limited evidence that air-polishing during maintenance therapy might cause a reduction in *A. actinomycetemcomitans*, but no difference is found in regard to other periodontal pathogens[26].

The aim of the present randomized controlled clinical trial was to evaluate the subgingival microbial community profile of patients affected by stage III–IV periodontitis treated via Guided Biofilm Therapy protocol[21], involving the use of subgingival air-polishing with erythritol + chlorhexidine powder and ultrasonic root surface debridement, versus ultrasonic debridement alone.

## Materials and methods

### Study design

This multicenter, single (examiner)-blinded, parallel arm randomized controlled clinical trial was conducted at the Section of Periodontics, School of Dentistry, Department of Surgical Specialties, Radiological Science and Public Health of the University of Brescia, within the ASST Spedali Civili di Brescia, Department of Odontostomatology, and at the Research Centre for the Study of Periodontal and Peri-implant Diseases, University of Ferrara. The protocol was reviewed and approved by the Ethics Committee of the University-Hospital of Brescia (CE: 2971) and the Ethics Committee of Area Vasta Emilia Centrale (protocol number: 83/2018/Disp/Unife) and the study conducted in accordance with the ethical principles of the Declaration of Helsinki. The study was registered on Clinical Trials.gov (NCT04264624).

### Patient selection and allocation

The study included systemically healthy adult participants (18–75 years, inclusive) affected by stage III–IV periodontitis. The participants were selected from the general population afferent to the aforementioned centers. The inclusion criteria for the study population were as follows:

- Diagnosis of stages III–IV periodontitis[27]
- At least 15 sites with probing depth (PD) 5–9 mm and positive to Bleeding on Probing (BoP)

The exclusion criteria for the study population were as follows:

- Pregnant or lactating
- Current or past (within 3 months of enrolment) medications that may influence periodontal conditions and/or interfere with healing following periodontal treatment (i.e., corticosteroids, calcium channel blockers)
- Non-surgical and/or surgical periodontal debridement within 3 months of enrolment, the use of systemically administered antibiotics within 3 months of enrolment
- Tumors or significant pathology of the soft or hard tissues of the oral cavity, or current radiotherapy or chemotherapy
- Chronic obstructive pulmonary disease and asthma
- History of allergy to erythritol or chlorhexidine
- Presence of orthodontic appliances

Smokers were included in the study.

All participants signed written informed consent before the beginning of the study. Randomized patient allocation to either test or control intervention was performed centrally using ad hoc software (R version 3.6.1, R Core Team (2020). R: A language and envi- ronment for statistical computing. R Foundation for Statistical Computing, Vienna, Austria. URL https://www.R-project.org/), using a blocked randomization scheme to achieve balanced treatment groups within centers.

### Interventions

At baseline (T0) and 3 months after treatment (T1) PD, clinical attachment level, gingival recession, BoP, and the presence of supragingival plaque (PII) were collected by a blinded examiner at 6 sites (mesio-buccal, mid-buccal, disto-buccal, mesio-lingual, mid-lingual, disto-lingual) for each tooth present. For each patient, all sites that showed PD 5–9 mm and were BoP-positive at T0 were identified as experimental sites.

Interventions were performed by the same two experienced calibrated operators (ES and CF).

Test group was identified according to the generated randomization table at the start of the session. After the application of a disclosing agent (Mira-2-Ton®, Hager & Werken, Duisburg, Germany) to guide plaque removal and achieve better biofilm removal[28], supra- and juxtagingival areas were air-polished (Airflow Prophylaxis Master, EMS, Nyon, Switzerland) with erythritol + chlorhexidine powder (PLUS powder, EMS, Nyon, Switzerland), followed by ultrasonic instrumentation for calculus removal with dedicated tip (PS tip, Airflow Prophylaxis Master, EMS, Nyon, Switzerland). This procedure is commonly known by practitioners with the name of Guided Biofilm Therapy (GBT). In patients allocated to Test intervention, experimental sites received subgingival biofilm removal with erythritol + chlorhexidine powder delivered via a specifically designed nozzle (Perioflow, EMS, Nyon, Switzerland) followed by subgingival ultrasonic instrumentation. In patients allocated to control intervention, experimental sites received subgingival ultrasonic instrumentation only. At the end of the session, the patients received oral hygiene instructions on manual toothbrushing and the use of interdental cleaning devices.

### Samples collection

Subgingival microbial samples were taken from the maxillary experimental site showing the deepest PD at T0. The same site was then sampled at T1. Prior to microbiological sampling, supragingival plaque was removed with a curette to avoid contamination of the sample with supragingival plaque. Sterile paper points size 30 or larger were inserted to the bottom of the periodontal pocket/sulcus and kept in place for 10 s. The samples were then placed in a sterile microcentrifuge tube and frozen until lab processing.

### Extraction of nucleic acids from samples

Each paper point was rehydrated in 0.3 ml of sterile RNase-free water and immediately vortexed 3 times for 30 s to detach nucleic acids. Then DNA and RNA were extracted from each sample by using the automatic Maxwell CSC Platform equipped with the HT Viral TNA kit (Promega, Madison, WI, USA), following the manufacturer’s instructions. Quality and concentration of extracted nucleic acids were assessed by spectrophotometric reading by using a Nanodrop at 260/280-nm wavelength (Thermo Fisher Scientific, Milan, Italy). The amplificability of extracted DNA was checked by polymerase chain reaction (PCR) amplification of bacterial 16S rRNA gene (pan-bacterial PCR, panB), as previously described[3]. Bi-distilled water samples containing 1mcg of DNA of non-related bacteria (e.g., *Bacillus* spp.) served as negative controls.

### Sample analysis

The microbial composition of each sample was analyzed by a real-time quantitative PCR (qPCR) microarray, providing profiling of microorganisms usually found in dental plaque and saliva (Microbial DNA qPCR Array for Oral Disease) (Qiagen, Hilden, Germany). Array assays are designed using the 16S rRNA gene as the target gene and individual primers and hydrolysis-probe detection, increasing specificity of each assay. Amplification was carried out in a Quant Studio 5 thermocycler (Thermo Fisher Scientific, Milan, Italy), providing simultaneous detection and quantification of 93 different microbial species, including putative and known periodontopathogens.

### Outcomes and statistical analysis

Primary outcome, as outlined in the first part of the present study[21], was the 3-month change in the number of sites with probing depth (PD) 5–9 mm and positive to bleeding upon probing (BoP), named “experimental sites.” The additional evaluation presented in this paper was the analysis of periodontal pathogens and other sub-gingival plaque bacteria sampled at one experimental site at baseline and 3 months following treatment. Sample size was estimated via Monte Carlo simulation. We assumed a proportion of NBCP at T1 of 40% in the control group and 1.7 odds-ratio of test group versus control. We used a fixed number of probed sites for every subject (*N* = 120, i.e., 6 sites for at least 20 teeth) and assumed a patient variance of 0.3. We simulated 1000 realizations of the event (PD < 5 mm and BoP-negative) at T1 using a binomial distribution and then modeled the simulated data using a GLMM logistic model with treatment (test vs control) as fixed effect and a single random component (patient intercept). The power is estimated as the proportion of simulations where the treatment effect was significant at the chosen 5% significant level. A sample size of 18 patients per group allowed for a power of at least 80%. Assuming a 10% attrition, we estimated a total sample size of 40 patients, equally randomized to the two treatment groups.

Statistical analyses were performed with GraphPad software. Kruskal–Wallis tests were performed to compare community composition in the groups, assuming *p* ≤ 0.05 as significant. Parametric and non-parametric Student’s *t*-test, Mann–Whitney, and ANOVA tests were used for group comparison, considering a *p* ≤ 0.05 as significant. For qPCR microarray data comparison, the Bonferroni correction for multiple comparisons was applied, assuming a corrected *p*_*c*_ ≤ 0.05 as statistically significant.

## Results

### Study design and patients groups

A total of 40 patients (20 for each center) were allocated to either test (*n* = 20) and control (*n* = 20) group. During the study, 2 patients in each group were excluded due to failure to attend to the appointments (*n* = 2) and need for antibiotic treatment due to other unrelated health issues (*n* = 2). Demographic data of the study population who completed the study are presented in Table 1. Groups were comparable for all considered variables.

**Table 1:** Patient characteristics in the test and control group

	Control (*N* = 18)	Test (*N* = 18)
Number of elements		
Mean (SD)	24.98 (3.16)	24.94 (2.41)
Median (Q1, Q3)	26.00 (23.00, 27.75)	25.50 (23.25, 26.75)
Smoker	3 (16.7%)	4 (22.2%)
Male	7 (38.9%)	11 (61.1%)
Age		
Mean (SD)	48.44 (9.31)	52.06 (10.17)
Median (Q1, Q3)	49.50 (42.50, 54.25)	53.00 (46.25, 60.00)
Number of experimental sites at T0		
Mean (SD)	59.89 (17.61)	49.56 (16.96)
Median (Q1, Q3)	60.00 (45.50, 76.25)	45.50 (38.00, 58.25)

*SD* standard deviation

#### Microbiological profile of the periodontal microbiome at T0

**Fig. 1:**
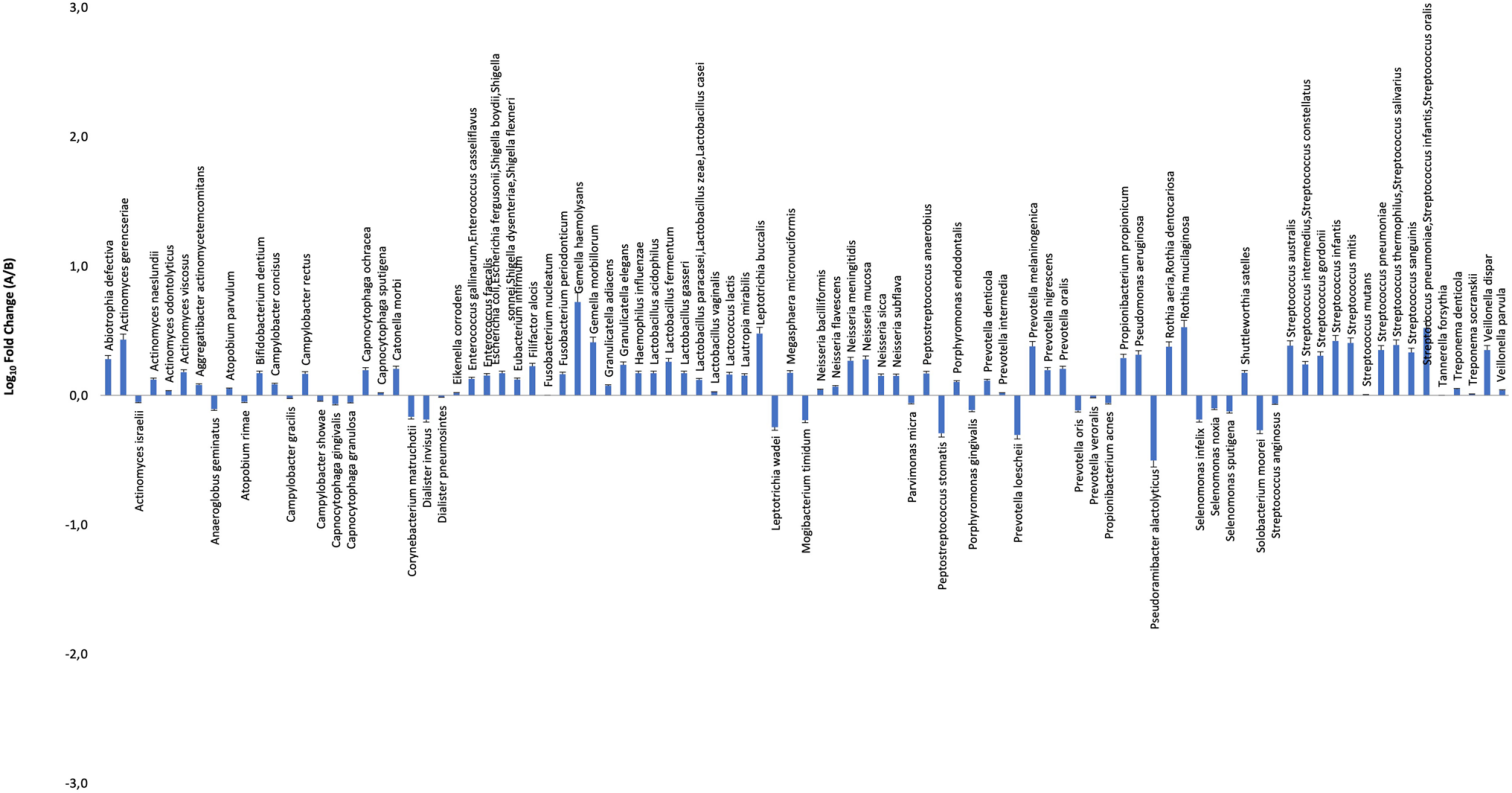
Microbiological profile of the periodontal microbiome at baseline, ratio between test group (**A**) and control group (**B**)

Microbiological results at T0 are reported in Table 2. Both groups showed a high presence of periodontopathogens, besides several other oral bacterial species. In both groups, *Campylobacter gracilis*/*rectus*, *Filifactor alocis*, *Fusobacterium nucleatum*, *Porphyromonas endodontalis/gingivalis*, *Tannerella forsythia*, and *Treponema denticola/sokranskii* were present at the highest level (> 3 Log-fold compared to negative controls), and *Actinomyces israelii/naeslundii*, *Parvimonas micra*, *Prevotella intermedia/oris*, and *Rothia dentocariosa/aeria* were detected with amounts > 2 Log-fold higher than negative controls. Some moderate differences were observed between the two groups (Fig. 1), regarding the presence of *Rothia* spp., *Streptococcus* spp., *Tannerella forsythia, Treponema denticola*, *Porphyromonas gingivalis*, and other less represented species. However, no statistically significant differences were evidenced in the basal periodontal samples between the two groups.

**Table 2:** Profile of the subgingival microbial population as detected by qPCR microarray at T0 and T1. Statistically significant *p* values are reported

Species	T0*		T1**				
	Test	Ctr	Test	Ctr	*p* value Ctr/test	*p* value Test T0/T1	*p* value Ctr T0/T1
*Abiotrophia defectiva*	0,78	0,49	1,34	0,71	**0.003**	**0.005**	
*Actinomyces gerencseriae*	1,22	0,69	0,33	0,85			
*Actinomyces israelii*	2,54	2,60	− 0,54	0,01		**0.018**	
*Actinomyces naeslundii*	2,05	1,97	0,34	0,83			
*Actinomyces odontolyticus*	1,56	1,53	0,30	0,50			
*Actinomyces viscosus*	1,67	1,44	0,67	1,11			
*Aggregatibacter actinomycetemcomitans*	0,46	0,46	0,13	0,44			
*Anaeroglobus geminatus*	1,19	1,45	− 0,03	− 0,09			
*Atopobium parvulum*	0,40	0,44	0,35	0,54			
*Atopobium rimae*	0,77	0,94	− 0,02	0,19			
*Bifidobacterium dentium*	0,23	0,06	0,21	0,59			
*Campylobacter concisus*	1,43	1,52	0,00	− 0,10			
*Campylobacter gracilis*	3,11	3,36	− 0,22	− 0,31			
*Campylobacter rectus*	3,42	3,17	− 0,93	− 0,72			
*Campylobacter showae*	1,62	1,74	0,45	0,09			
*Capnocytophaga gingivalis*	1,45	1,75	1,15	0,23			
*Capnocytophaga granulosa*	1,40	1,37	0,46	0,38			
*Capnocytophaga ochracea*	2,08	1,84	− 0,19	0,51			
*Capnocytophaga sputigena*	1,45	1,36	0,43	0,00	**0.027**	**0.009**	
*Catonella morbi*	2,13	2,01	− 0,69	0,14		**0.004**	
*Corynebacterium matruchotii*	2,18	2,43	0,37	0,71			
*Dialister invisus*	1,39	1,68	− 0,19	− 0,01			
*Dialister pneumosintes*	1,67	1,63	− 0,41	-0,10			
*Eikenella corrodens*	1,59	1,70	0,91	0,27			
*Enterococcus gallinarum,Enterococcus casseliflavus*	0,23	0,17	0,21	0,58			
*Enterococcus faecalis*	0,23	0,14	0,21	0,51			
*Escherichia coli*, *Escherichia fergusonii*, *Shigella boydii*, *Shigella sonnei*, *Shigella dysenteriae*, *Shigella flexneri*	0,23	0,06	0,21	0,59			
*Eubacterium infirmum*	0,36	0,31	0,18	0,62			
*Filifactor alocis*	3,31	3,30	− 1,06	− 0,42		**0.02**	**0.03**
*Fusobacterium nucleatum*	4,25	4,43	− 0,24	− 0,28			
*Fusobacterium periodonticum*	1,19	0,91	0,46	0,63			
*Gemella haemolysans*	1,73	0,81	0,26	0,58			
*Gemella morbillorum*	2,53	2,04	− 0,07	− 0,26			
*Granulicatella adiacens*	1,45	1,56	0,56	0,49			
*Granulicatella elegans*	1,46	1,04	0,27	0,46			
*Haemophilus influenzae*	0,23	0,06	0,21	0,59			
*Lactobacillus acidophilus*	0,23	0,06	0,21	0,59			
*Lactobacillus fermentum*	0,37	0,06	0,07	0,59			
*Lactobacillus gasseri*	0,23	0,06	0,21	0,59			
*Lactobacillus paracasei*, *Lactobacillus zeae*, *Lactobacillus casei*	0,23	0,18	0,21	0,47			
*Lactobacillus vaginalis*	0,23	0,27	0,21	0,48			
*Lactococcus lactis*	0,23	0,13	0,30	0,65			
*Lautropia mirabilis*	1,83	1,72	1,37	0,75	**0.038**	**0.005**	
*Leptotrichia buccalis*	1,22	0,51	0,11	0,71			
*Leptotrichia wadei*	0,51	0,98	0,19	0,44			
*Megasphaera micronuciformis*	0,63	0,32	0,00	0,41			
*Mogibacterium timidum*	1,65	2,01	− 0,59	− 0,11			
*Neisseria bacilliformis*	0,78	0,85	0,04	0,16			
*Neisseria flavescens*	0,80	0,74	0,75	0,43			
*Neisseria meningitidis*	0,44	0,06	0,30	0,59			
*Neisseria mucosa*	1,38	1,00	1,00	0,58	**0.025**	**0.053**	
*Neisseria sicca*	0,98	0,83	0,71	0,30			
*Neisseria subflava*	1,09	0,84	0,92	0,50			
*Parvimonas micra*	2,41	2,77	− 0,11	− 0,13			
*Peptostreptococcus anaerobius*	0,23	0,13	0,21	0,52			
*Peptostreptococcus stomatis*	0,68	1,15	0,14	0,10			
*Porphyromonas endodontalis*	3,30	3,47	− 1,00	− 0,08		**0.018**	
*Porphyromonas gingivalis*	2,18	3,96	− 0,49	− 0,84			
*Prevotella denticola*	1,34	1,31	− 0,29	0,11			
*Prevotella intermedia*	1,76	2,86	− 0,03	− 0,01			
*Prevotella loescheii*	1,68	2,12	0,46	0,11			
*Prevotella melaninogenica*	2,22	1,62	0,05	0,49			
*Prevotella nigrescens*	2,66	2,37	− 0,75	− 0,07			
*Prevotella oralis*	1,14	0,94	− 0,09	0,84			
*Prevotella oris*	2,55	3,40	− 0,17	− 0,66			
*Prevotella veroralis*	0,41	0,52	0,24	0,27			
*Propionibacterium acnes*	1,84	1,90	− 0,03	0,44			
*Propionibacterium propionicum*	1,36	1,06	0,52	0,71			
*Pseudomonas aeruginosa*	0,48	0,06	− 0,04	0,59			
*Pseudoramibacter alactolyticus*	0,81	1,62	0,04	0,16			
*Rothia aeria*, *Rothia dentocariosa*	3,16	2,77	0,16	0,63			
*Rothia mucilaginosa*	2,08	1,44	0,01	1,42			
*Selenomonas infelix*	1,92	2,26	− 0,38	− 0,28			
*Selenomonas noxia*	1,72	1,84	− 0,07	0,22			
*Selenomonas sputigena*	1,50	1,79	− 0,52	− 0,20		**0.033**	
*Shuttleworthia satelles*	0,40	0,30	0,17	0,35			
*Solobacterium moorei*	0,85	1,38	0,11	0,00			
*Streptococcus anginosus*	1,01	1,09	− 0,24	0,10			
*Streptococcus australis*	2,20	1,80	0,44	0,49			
*Streptococcus intermedius*, *Streptococcus constellatus*	2,65	2,46	− 0,30	− 0,01			
*Streptococcus gordonii*	1,31	1,08	0,05	0,46			
*Streptococcus infantis*	2,86	2,44	0,32	0,56			
*Streptococcus mitis*	2,60	2,00	0,37	0,51			
*Streptococcus mutans*	0,23	0,36	0,21	0,40			
*Streptococcus pneumoniae*	2,92	2,57	0,16	0,41			
*Streptococcus thermophilus*, *Streptococcus salivarius*	1,38	0,99	0,12	0,72			
*Streptococcus sanguinis*	2,56	2,22	0,41	0,78			
*Streptococcus pneumoniae*, *Streptococcus infantis*, *Streptococcus oralis*	3,44	2,92	0,09	0,43			
*Tannerella forsythia*	3,59	4,21	− 0,86	− 0,99		**0.029**	**0.001**
*Treponema denticola*	2,98	3,19	− 0,90	− 0,42		**0.041**	
*Treponema socranskii*	2,58	2,92	− 0,59	− 0,38		**0.021**	**0.042**
*Veillonella dispar*	0,73	0,25	− 0,08	0,87			
*Veillonella parvula*	1,58	1,66	0,48	0,50

#### Microbiological profile of the periodontal microbiome at T1

**Fig. 2:**
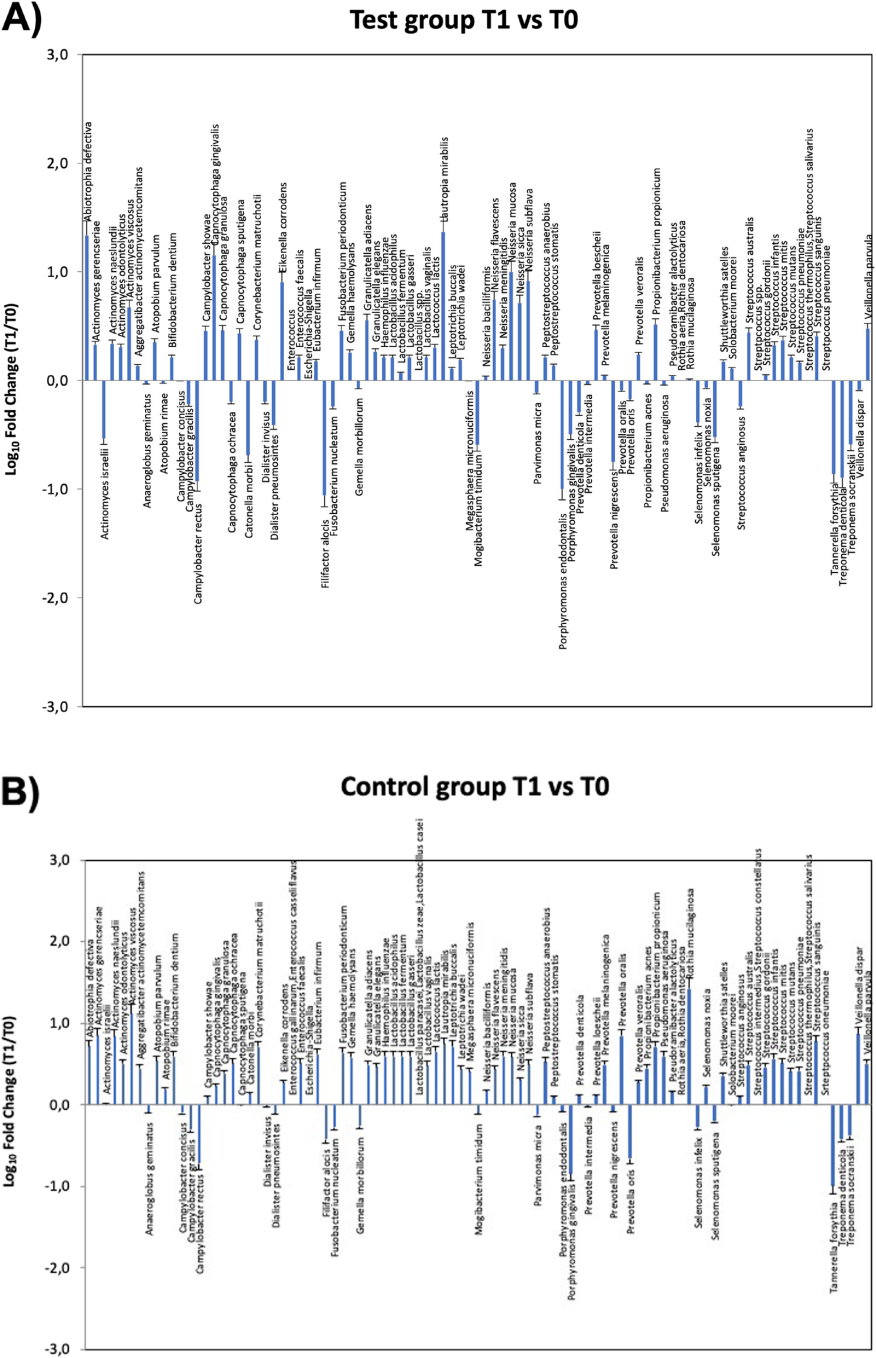
Microbiological profile of the periodontal microbiome at baseline for test group (**A**) and control group (**B**), ratio between T1 and T2

**Fig. 3:**
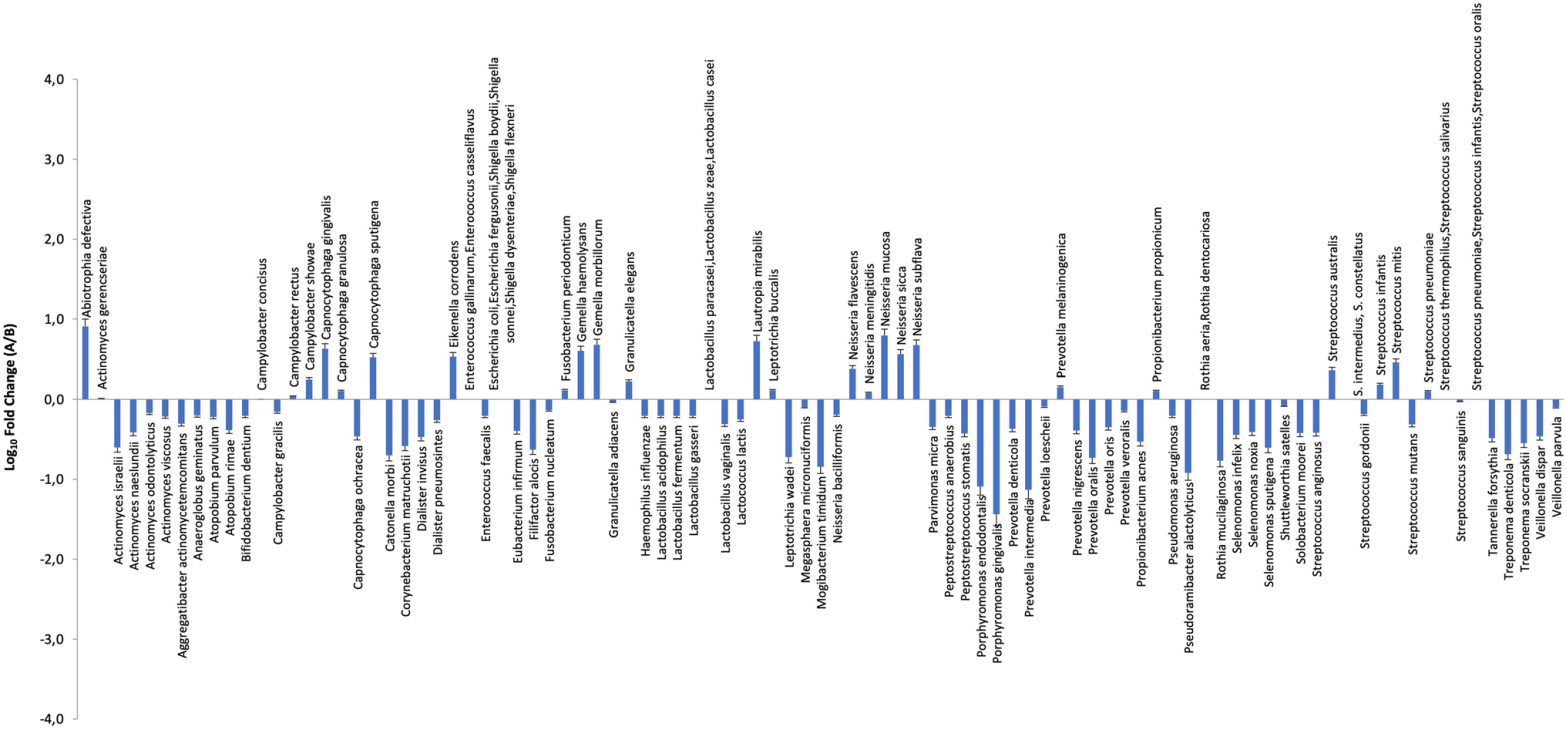
Microbiological profile of the periodontal microbiome at T1, ratio between test group (**A**) and control group (**B**)

After treatment, the analysis of the periodontal microbiome profile showed the presence of the same species detected at the basal level, although quantitative variations were observed in both groups in the amount of both periodontopathogens and commensal/protective bacterial species (Fig. 2). In particular, statistical significance was observed in the test group before and after treatment relatively to the increase of *Abiotropha defectiva* (*p* = 0.005)*, Capnocytophaga sputigena* (*p* = 0.009), and *Lautropia mirabilis* (*p* = 0.005), together with the decrease of *Actinomyces israelii* (*p* = 0.018), *Catonella morbi* (*p* = 0.004), *Filifactor alocis* (*p* = 0.02), *Porphyromonas endodontalis* (*p* = 0.018), *Selenomonas sputigena* (*p* = 0.033), *Tannerella forsythia* (*p* = 0.029), *Treponema denticola* (*p* = 0.041), and *Treponema socranskii* (*p* = 0.021). In the control group, statistical significance was found only in the decrease of *Filifactor alocis* (*p* = 0.03), *Tannerella forsythia* (*p* = 0.001), and *Treponema socranskii* (*p* = 0.042) (Fig. 3). Despite the lack of significant difference in the type of bacteria detected in the two groups at T0 and T1, diverse quantitative alterations were observed according to the type of treatment (Fig. 3). Compared to the control group, test showed a greater increase of species including *Abiotropha defectiva*, *Capnocytophaga sputigena*, *Lautropia mirabilis*, *Neisseria mucosa*, and a concomitant more pronounced decrease of several species, including *Actinomyces israelii*, *Catonella morbi*, *Filifactor alocis*, *Mogibacterium timidum*, *Porphyromonas endodontalis*, *Selenomonas sputigena*, *Tannerella forsythia*, *Treponema denticola* and *socranskii*. Most samples where periodontopathogens resulted decreased showed in parallel significant increases of bacteria including *Abiotropha defectiva*, *Lautropia mirabilis*, and *Neisseria mucosa.* Increases were observed also regarding *Capnocytophaga sputigena*, though they did not result statistically significant.

### Microbiological profile: patient and site characteristics

**Fig. 4:**
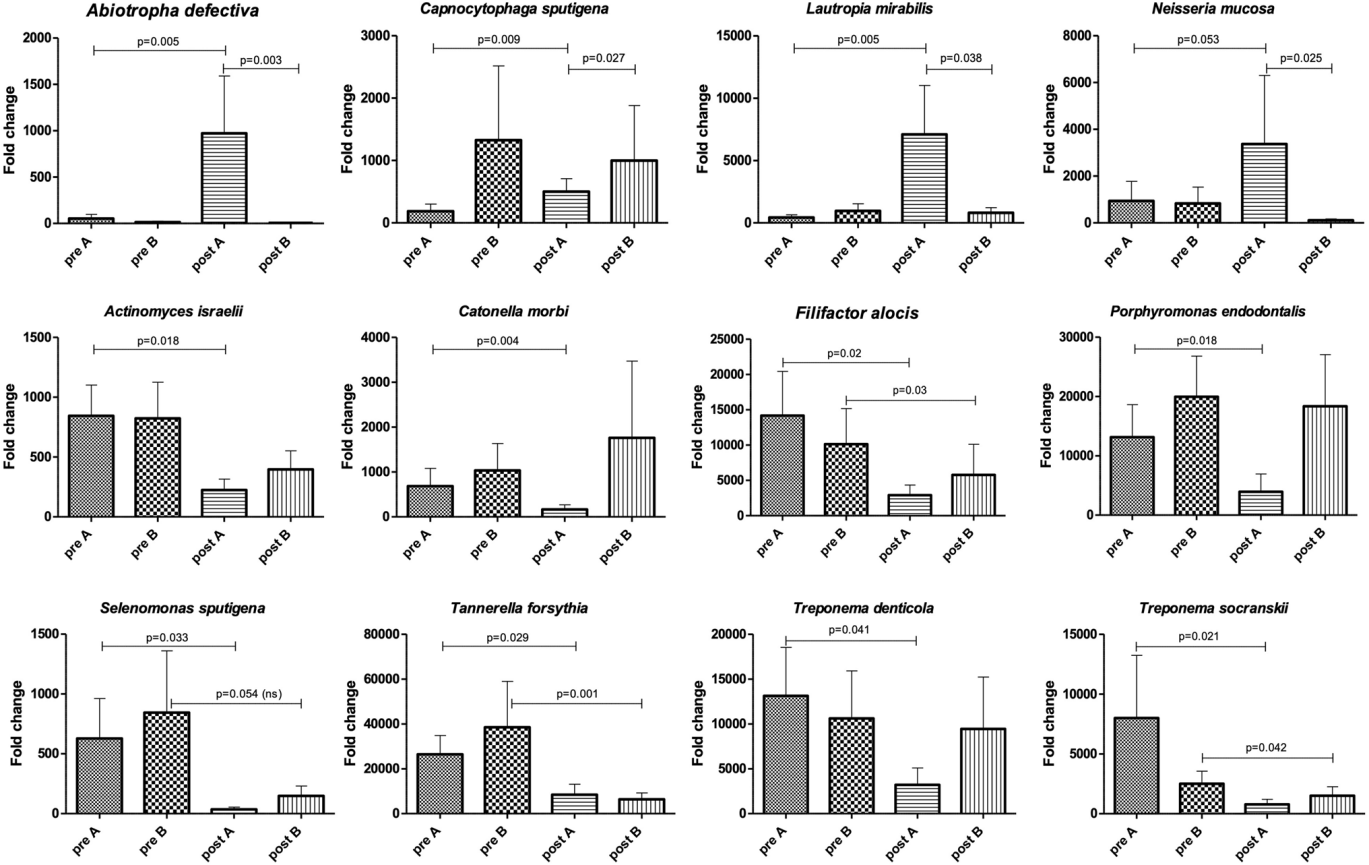
Post-treatment fold change of bacterial species showing a statistically significant quantitative alterations for test group (**A**) and control group (**B**)

**Fig. 5:**
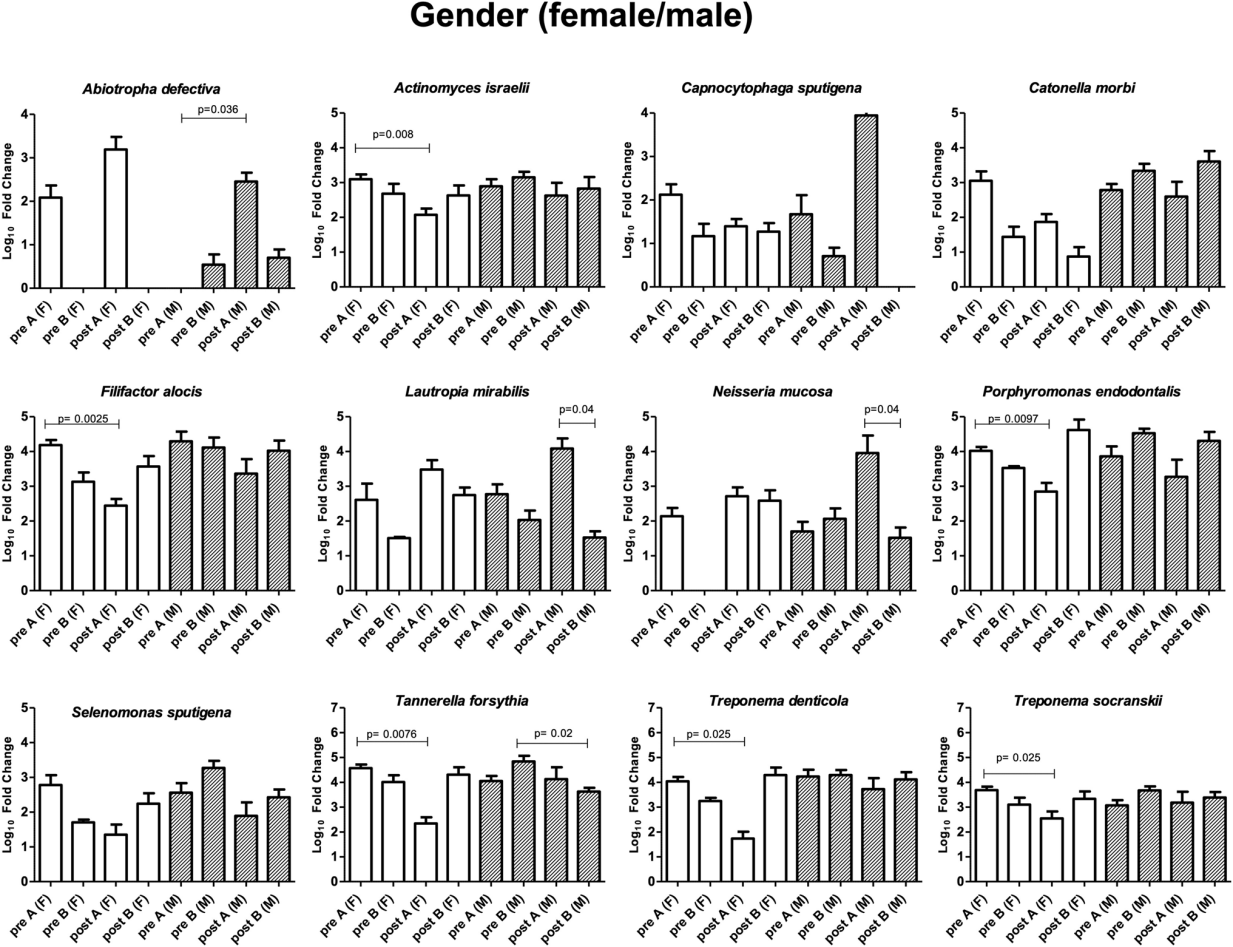
Post-treatment fold change of bacterial species showing a statistically significant quantitative alterations for test group (**A**) and control group (**B**)—gender sub-analysis

**Fig. 6:**
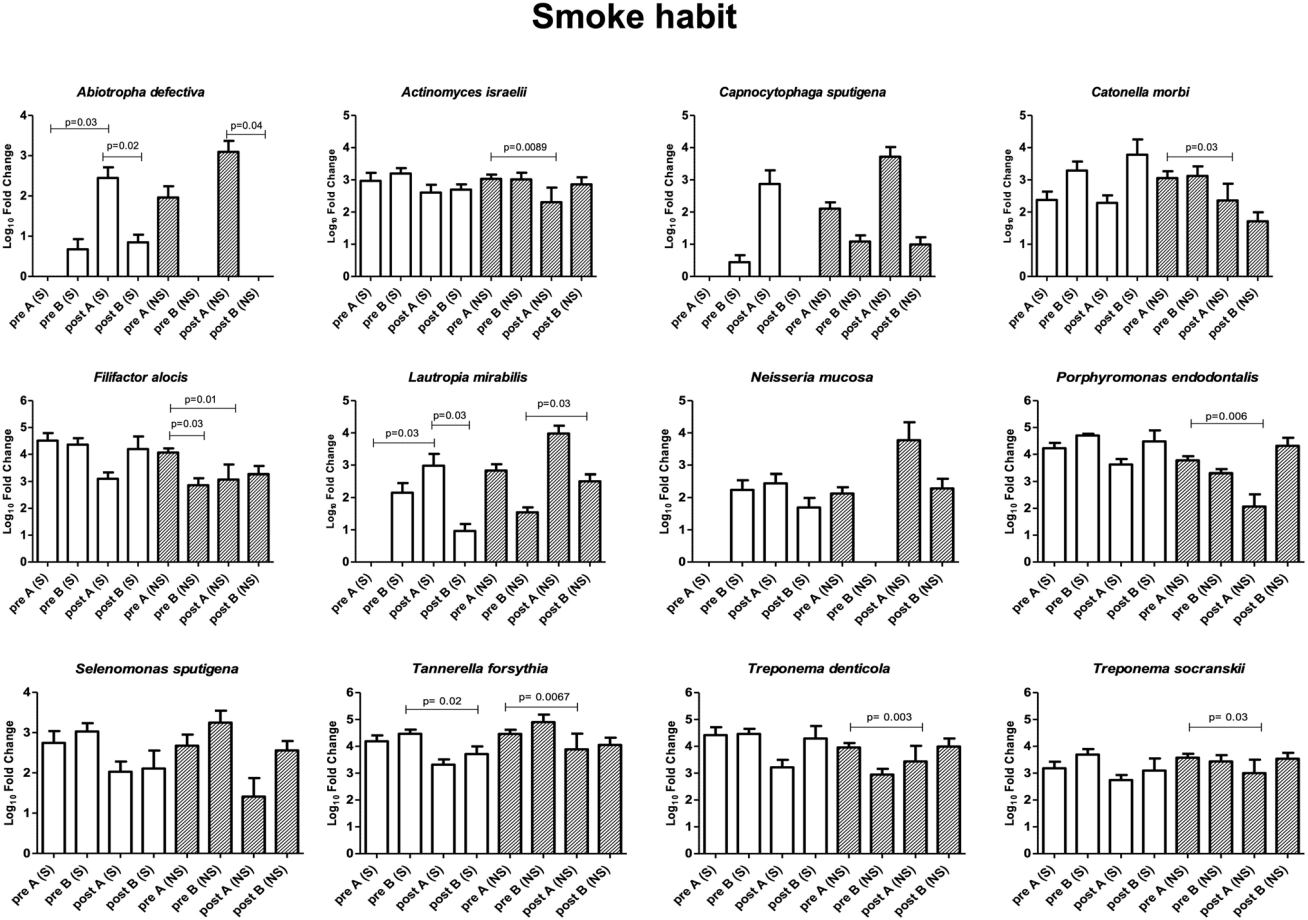
Post-treatment fold change of bacterial species showing a statistically significant quantitative alterations for test group (**A**) and control group (**B**)—Smoking habit sub-analysis

**Fig. 7:**
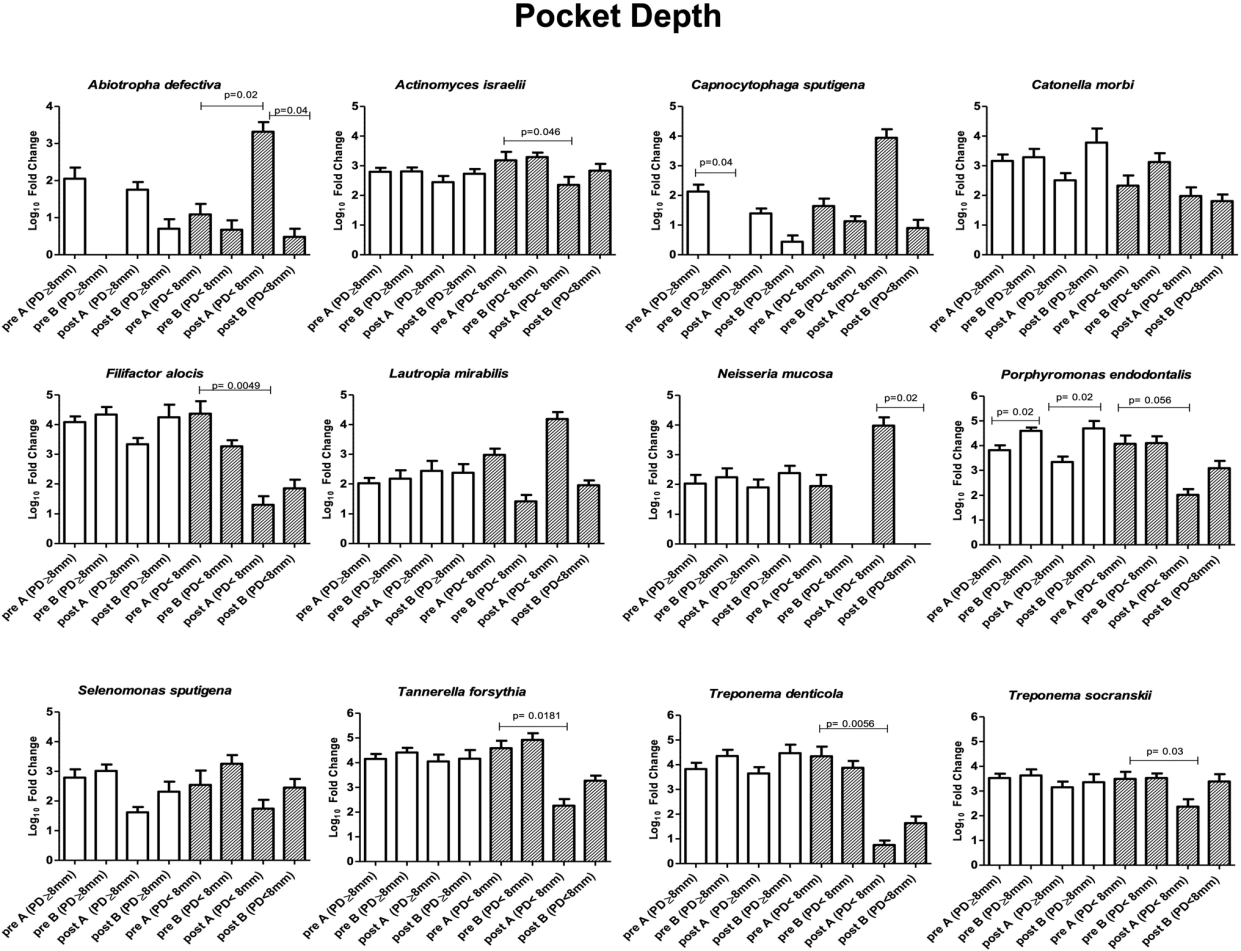
Post-treatment fold change of bacterial species showing a statistically significant quantitative alterations for test group (**A**) and control group (**B**)—pocket depth sub-analysis

**Fig. 8:**
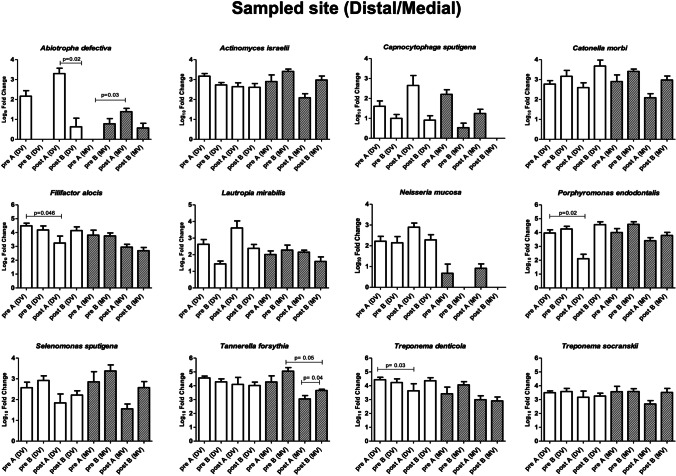
Post-treatment fold change of bacterial species showing a statistically significant quantitative alterations for test group (**A**) and control group (**B**)—distal/medial sites sub-analysis

**Fig. 9:**
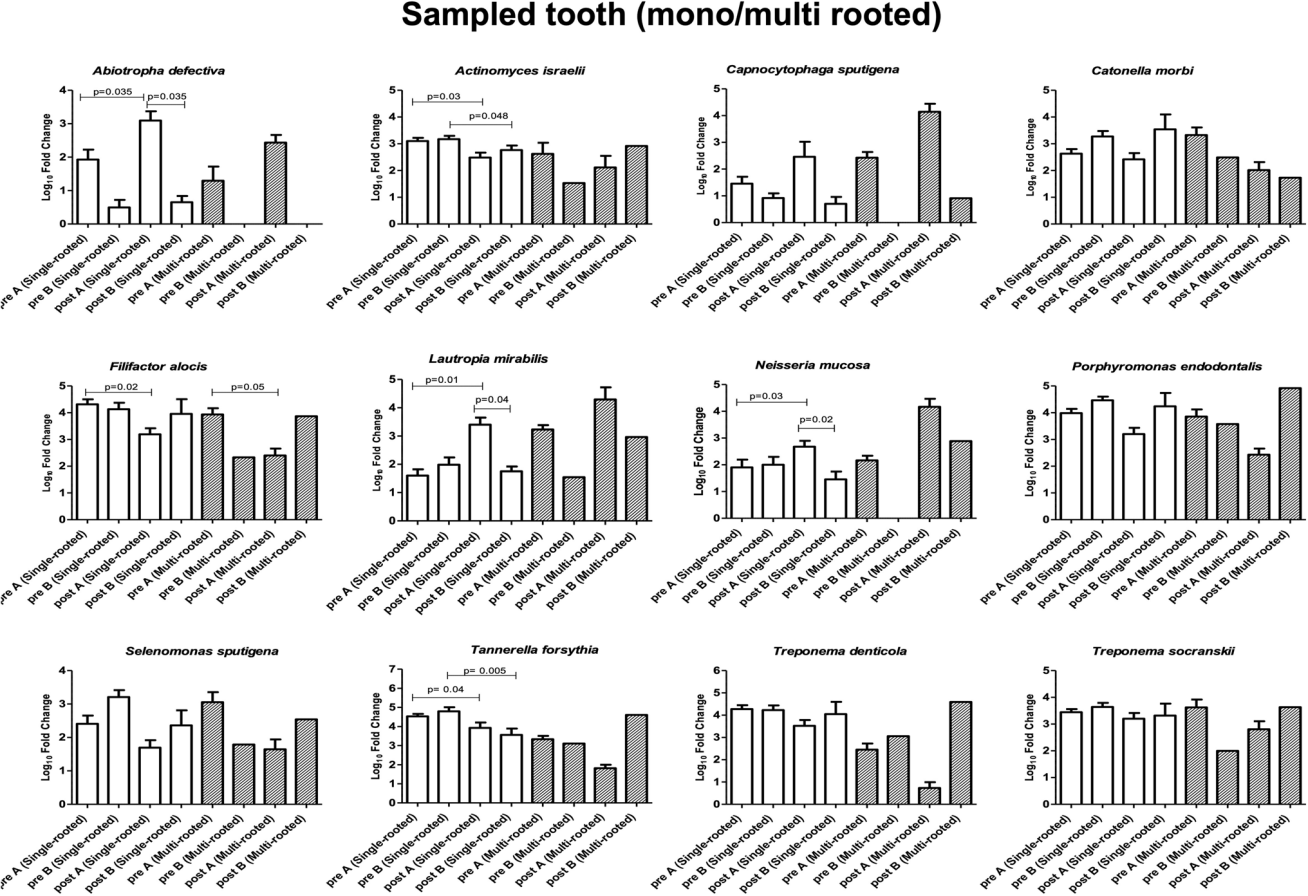
Post-treatment fold change of bacterial species showing a statistically significant quantitative alterations for test group (**A**) and control group (**B**)—tooth type sub-analysis (mono or multi rooted)

Focusing on the bacteria that showed significant alterations (Fig. 4), variations were observed dependent on epidemiological features or on the characteristics of the sampled site (Figs. 5–9). Bacteria possibly associated with periodontitis (*Actinomyces israelii*, *Filifactor alocis*, *Porphyromonas endodontalis*, *Tannerella forsythia*, *Treponema denticola*, and *Treponema socranskii*) were significantly diminished mostly in female gender (Fig. 5) and in non-smokers (Fig. 6) of group A (0.003 ≤ *p* ≤ 0.03), rather than in male gender and in formers smokers, where only the species *Tannerella forsythia* in the control group (*p* = 0.02) appeared significantly diminished after treatment. Significant differences were also observed relative to the depth of dental pockets (higher or lower than 8 mm); in detail, *Actinomyces israelii*, *Filifactor alocis*, *Porphyromonas endodontalis*, *Tannerella forsythia*, and *Treponema denticola/socranskii* appeared more decreased in the subjects with pocket depth < 8 mm of group A (0.005 ≤ *p* ≤ 0.05), compared to those with more profound pockets and those included in the control group, who only showed a significant decrease in *Porphyromonas endodontalis* (*p* = 0.02) (Fig. 7). Less evident differences were detected according to the tooth type (single-rooted *vs* multi-rooted) and the sampled site (distal *vs* medial). *Actinomyces israelii* and *Tannerella forsythia* were in fact the only bacterial species significantly diminished in single rooted teeth of both test and control group (Fig. 8); it also appeared significantly decreased in medial sites of both groups, whereas distal sites of the test group showed significant decreases also in the amount of *Porphyromonas endodontalis* and *Treponema denticola* (Fig. 9)*.*

The results obtained on the whole periodontal microbiome are summarized in Supplementary Figs. 1–10, showing the microbiological profile in the sub-groups of patients, at the basal and follow-up level, subdivided accordingly with treatment type (A = test, B = control). The clinical results are reported in the previous part of this paper[15].

## Discussion

**Fig. 10:**
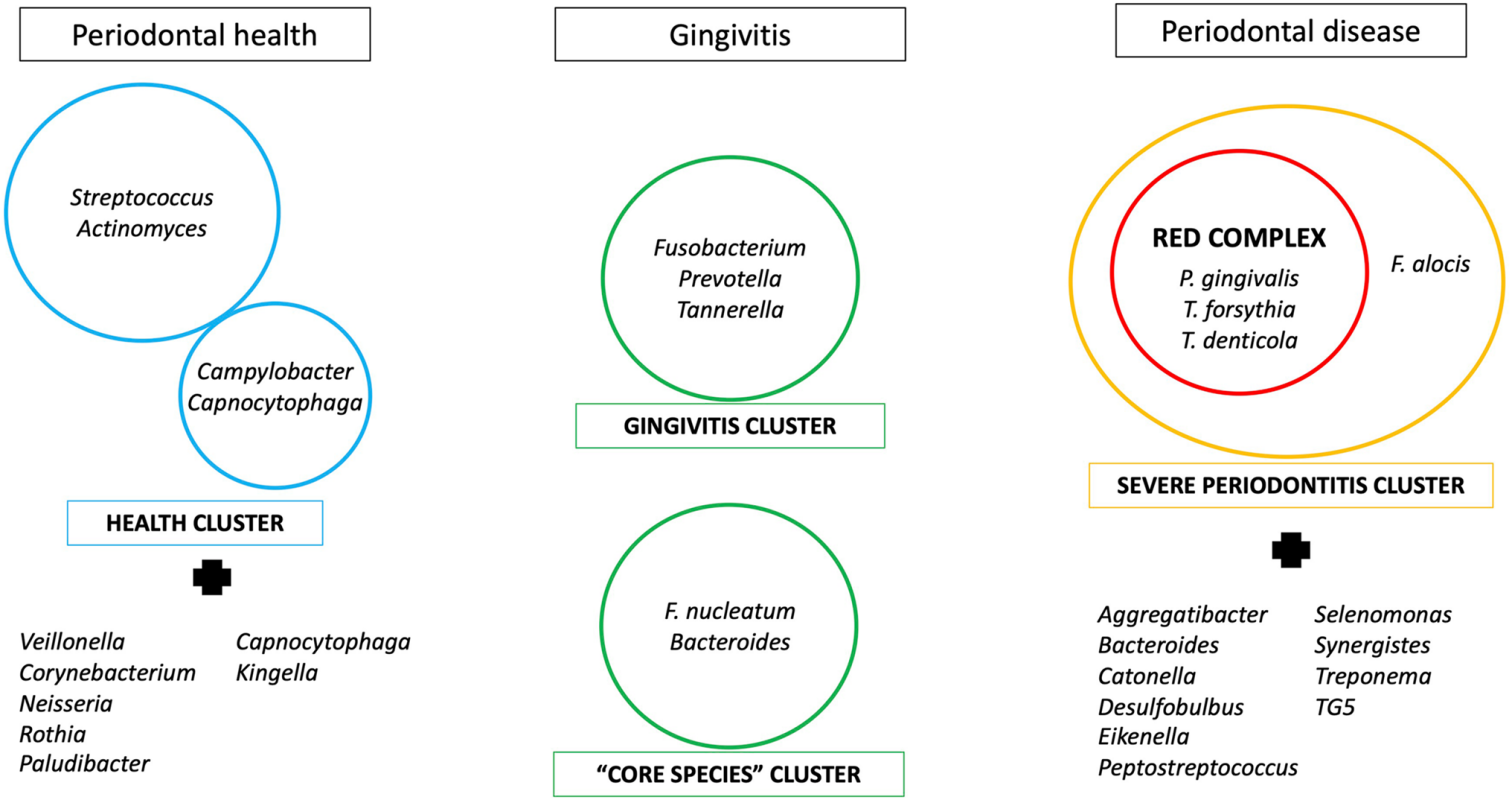
Bacterial clusters associated with periodontal health, gingivitis, and periodontal disease

Periodontal disease, an inflammatory destructive disease of the periodontium caused by a disbyotic microbial community, is associated with specific presence and abundance of bacteria in the subgingival biofilm, namely those belonging to the so-called red complex, including *Porphyromonas gingivalis*, *Tannerella forsythia*, and *Treponema denticola*[29]. However, recent studies including large numbers of periodontal samples showed significant differences in the oral microbiome between periodontal patients and healthy subjects, not only limited to the mentioned bacteria[15]. In particular, *Treponema*, *TG5*, *Desulfobulbus*, *Catonella*, *Bacteroides*, *Aggregatibacter*, *Peptostreptococcus*, *Eikenella*, *Selenomonas*, and *Synergistes* emerged as periodontitis biomarkers, while *Veillonella*, *Corynebacterium*, *Neisseria*, *Rothia*, *Paludibacter*, *Capnocytophaga*, and *Kingella* are now considered signatures of a healthy periodontium[15, 30]. Moreover, new species has emerged as significantly associated with periodontitis, such the Gram-positive anaerobic *Filifactor alocis*, with peculiar characteristics in terms of virulence potential and capacity to influence the oral microbiome[13]. Interestingly, molecular profiling of subgingival plaque samples, based on 16S rRNA gene sequencing, suggests the existence of different microbial clusters associated with disease[17]. The cluster characterized by the presence of *Fusobacterium*, *Prevotella*, and *Tannerella* species seems usually associated with gingivitis, while the cluster containing the red-complex species and the newly emerged *Filifactor alocis* seems to be strongly associated with severe periodontitis. On the other side, also bacterial species associated with periodontal and oral health can be subdivided in two clusters: a large group consisting mainly of *Streptococcus* and *Actinomyces* species and a smaller group consisting of *Campylobacter* and *Capnocytophaga* species[31]. Last, another cluster (named “core species”) includes mainly *Fusobacterium nucleatum* and *Bacteroides* spp., and appears to be mostly associated with gingivitis, which is considered the transitional stage from health to disease (Fig. 10). The transition from health to periodontitis may be determined by the evolution of such microbial clusters, each representing a different stage of microbiome dysbiosis, related to the local environment, metabolic exchanges, and local inflammatory stimuli.

Based on these premises, our study aimed to define the modulation of the periodontal microbiome in response to subgingival ultrasonic debridement with or without the adjunctive use of air-polishing with erythritol powder + chlorhexidine. This is the second part of a study previously published by the same research group, reporting the clinical outcomes of the proposed treatments[21]. In the first part of the study, we observed that the adjunctive air-polishing did not seem to provide any significant advantage in terms of pocket closure at 3 months after the treatment, in line with the available literature[23–25]. However, it was of interest for the researchers to investigate whether the subgingival application of known antibacterial agents such as chlorhexidine and erythritol, conveyed through a jet of water that could reach a bigger area of the pocket, might help to shift the microbiota from a condition of dysbiosis to healthy eubiosis, possibly preventing the recurrence of the disease.

Willing to compare the amount of specific periodontitis-related and health-related bacteria before and after treatment, the microbiological profile of the periodontal niche was analyzed by qPCR microarray, allowing to quantitate the individual potential periodontopathogens, contrary to what could be obtained by 16S rRNA sequencing technique, which only provide relative abundance data.

The analysis at baseline (T0) showed, as expected, a high prevalence and amount of known and putative periodontal bacteria in the subgingival microbiome of the enrolled subjects: besides the classical periodontopathogens, including the genera *Fusobacterium*, *Porphyromonas*, *Prevotella*, *Tannerella*, and *Treponema*, other species were found highly prevalent, highlighting their potential role in periodontitis. In particular, *Filifactor alocis* was abundant in both groups of treated subjects, and the treatment induced its significant decrease, confirming its putative role as a periodontal species. Other significantly diminished genera after treatment included *Porphyromonas*, *Tannerella*, and *Treponema*, confirming their role in periodontitis and the association between their decrease and the success of treatment.

Statistically significant modulations were mostly observed in test group, rather than control group, suggesting the ability of erythritol + chlorhexidine powder treatment to influence more deeply the recovery of a healthy periodontal microbiome. Chlorhexidine is a well-known antimicrobial, and erythritol seems to be able to suppress the growth of *P. gingivalis* in vitro[32]. The significant decrease in *Porphyromonas* spp. was observed in the GBT group in the present study is somehow in line with Park et al. (2018)[24], who found the additional application or erythritol powder in periodontal pockets during non-surgical treatment lowered the relative expression of *P. gingivalis* at 1 month after treatment. The reduction of these species is important for the prevention of further alveolar and periodontal destruction[33]. However, Park et al. (2018)[24] failed to show any significant difference in the post-treatment expression of the members of the red complex, *T. forsythia* and *T. denticola.* In another study from Jentsch et al. (2020)[23], significantly greater reductions of *Tannerella forsythia* and *Treponema denticola* counts were observed at 6 months after the treatment with erythritol powder, when compared to hand and sonic instrumentation. This difference could be explained by the shorter observation period in Park et al. (2018)[24] and the present study, and a major difference in protocol: in Jentsch et al. (2020)[23], patients received a session of supportive periodontal therapy involving the subgingival application of erythritol + chlorhexidine, which could have further reduced the periodontal pathogens. However, whether there is a microbiological benefit in multiple applications of subgingival air-polishing is still uncertain. A study by Müller et al. (2014)[26] failed to observe any significant microbial advantage after 12 months of 3 monthly maintenance periodontal therapy with erythritol powder, with the only exception in the reduction of *Aggregatibacter actinomycetemcomitans.*

Absence of disease does not only involve the reduction of pathogenic bacteria, but also the re-establishment of a healthy microbiota[1]. Therefore, the present study also focused on bacterial species associated to periodontal health. Genera showing evident variations (some of which statistically significant) after treatment included *Abiotrophia*, *Actinomyces*, *Capnocytophaga*, *Catonella*, *Lautropia*, *Neisseria*, and *Selenomonas.* Among those bacteria, a protective role has been suggested for *Actinomyces*, *Capnocytophaga*, *Lautropia*, and *Neisseria*, which have been attributed a role in the maintenance of eubiosis at the periodontal level[31, 34]. Of note, most of these genera resulted increased after treatment, in a significant way especially for the test group, supporting the idea that the success of treatment could be associated with a rebalance of periodontal microbiome. The process leading from dysbiosis to eubiosis is still not completely clear, but it is likely to involve the removal of the initial stressors such as alterations to the effectiveness of the immune response and activity of pathogenic bacterial species able to manipulate the overall bacterial population[35]. When the stressors are removed, health can be re-established and maintained. The significant reduction in periodontal pathogens and the increased in health-related species observed in all the subjects of the present study, and the extremely positive clinical results reported in the previous part of this paper[21] seem to confirm that a change toward minimally invasive periodontal procedures is not only possible but also advantageous. Root surface debridement is sufficient for biofilm management and, as reported in literature, not only is a gentler form of instrumentation compared to root planing, but also more comfortable, simple, time saving, and equally effective[20, 36].

To assess the possible impact of the different epidemiological or disease characteristics of the enrolled subjects on the response to treatment and consequent microbiome remodulation, we stratified the results accordingly to such features and analyzed the microbial significance separately. The enrolled subjects were thus stratified according to gender, smoking habit, and sampling performed in single-rooted or multi-rooted teeth, periodontal pockets greater or less than 8 mm of depth, and distal or medial tooth sites. The collected data showed that female gender and non-smokers could get better response in terms of microbial remodulation of the periodontal profile, compared to males and smokers. While the immunosuppressive nature of tobacco is well known[37], the male/female difference could be due to gender-associated differences in the oral microbiota, as it appears that males might present a higher prevalence of *Porphyromonas* and *Capnocytophaga* spp.[38]. In addition, the depth of the dental pockets at the enrollment also influenced the significance of microbial rebalance, as smaller pockets (< 8 mm) responded better to treatment, especially in the test group. This could be explained by the limited ability of the subgingival nozzle to penetrate deeper in the pocket, due to its thickness. Other features, such as distal or medial sampling site and single- or multi-rooted teeth, influenced less the microbiological response toward the type of treatment, with some significant shifts only on single-rooted teeth, probably due to the easier access for debridement. This is in agreement with the first part of this study[21], in which the probability of transforming an experimental site to a close non-bleeding pocket resulted higher for single-rooted teeth, and the deeper the pocket the lower the probability of it becoming a healthy sulcus. Interestingly, smoking status did not significantly influence the clinical outcome. Despite a more significant microbiological amelioration in the test group compared to the control group, no significant clinical differences in the outcome of patients were reported in the two groups in the first part of this study[21]. This may be due to the relatively low number of assayed subjects, and perhaps a more prolonged period of observation and/or a higher number of subjects may help to elucidate whether the better balance of the periodontal microbiome in the test group could be associated with a prolonged health of the treated periodontium.

## Conclusions

In conclusion, the GBT treatment including the addition of erythritol-chlorhexidine powder was accompanied by a shift of the periodontal microbiome toward a more eubiotic condition compared to a conventional treatment lacking such addition.

## Supplementary Information

Below is the link to the electronic supplementary material.

Supplementary file1 (PDF 825 KB)

## Data Availability

Data available on request from the authors.
