# Supplementary material for: Efficacy of the additional use of subgingival air-polishing with erythritol powder in the treatment of periodontitis patients: a randomized controlled clinical trial. Part II: effect on sub-gingival microbiome
Source: Clin Oral Investig. 2022 Dec 20;27(6):2547–63. doi: 10.1007/s00784-022-04811-4 (PMC10264538; doi:10.1007/s00784-022-04811-4)
Supplement: Supplementary file 1 — Supplementary file1 (PDF 825 KB) [file 784_2022_4811_MOESM1_ESM.pdf]

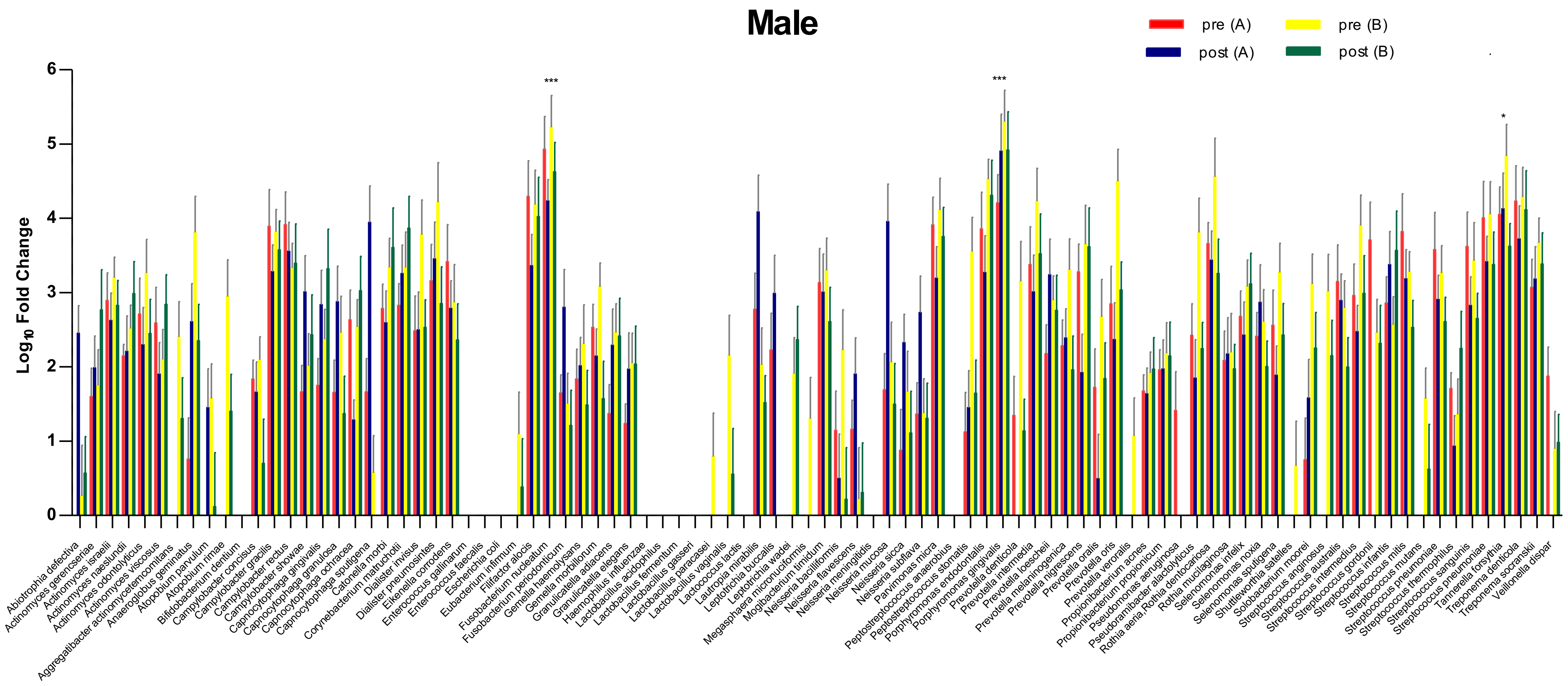

Male
pre (A)
pre (B)
post (A)
post (B)
Log10 Fold Change
0
1
2
3
4
5
6
***
***
*
Abiotrophia defectiva
Actinomyces gerencseriae
Actinomyces israelii
Actinomyces naeslundii
Actinomyces odontolyticus
Actinomyces viscosus
Aggregatibacter actinomycetemcomitans
Anaeroglobus geminatus
Atopobium parvulum
Atopobium rimae
Bifidobacterium dentium
Campylobacter concisus
Campylobacter gracilis
Campylobacter rectus
Campylobacter showae
Capnocytophaga gingivalis
Capnocytophaga granulosa
Capnocytophaga ochracea
Capnocytophaga sputigena
Catonella morbi
Corynebacterium matruchotii
Dialister invisus
Dialister pneumosintes
Eikenella corrodens
Enterococcus gallinarum
Enterococcus faecalis
Escherichia coli
Eubacterium infirmum
Filifactor alocis
Fusobacterium nucleatum
Fusobacterium periodonticum
Gemella haemolysans
Gemella morbillorum
Granulicatella adiacens
Granulicatella elegans
Haemophilus influenzae
Lactobacillus acidophilus
Lactobacillus fermentum
Lactobacillus gasseri
Lactobacillus paracasei
Lactobacillus vaginalis
Lactococcus lactis
Lautropia mirabilis
Leptotrichia buccalis
Leptotrichia wadei
Megasphaera micronuciformis
Mogibacterium timidum
Neisseria bacilliformis
Neisseria flavescens
Neisseria meningitidis
Neisseria mucosa
Neisseria sicca
Neisseria subflava
Parvimonas micra
Peptostreptococcus anaerobius
Peptostreptococcus stomatis
Porphyromonas endodontalis
Porphyromonas gingivalis
Prevotella denticola
Prevotella intermedia
Prevotella loescheii
Prevotella melaninogenica
Prevotella nigrescens
Prevotella oralis
Prevotella oris
Prevotella veroralis
Propionibacterium acnes
Propionibacterium propionicum
Pseudomonas aeruginosa
Pseudoramibacter alactolyticus
Rothia aeria,Rothia dentocariosa
Rothia mucilaginosa
Selenomonas infelix
Selenomonas noxia
Selenomonas sputigena
Shuttleworthia satelles
Solobacterium moorei
Streptococcus anginosus
Streptococcus australis
Streptococcus intermedius
Streptococcus gordonii
Streptococcus infantis
Streptococcus mitis
Streptococcus mutans
Streptococcus pneumoniae
Streptococcus thermophilus
Streptococcus sanguinis
Streptococcus pneumoniae
Tannerella forsythia
Treponema denticola
Treponema socranskii
Veillonella dispar

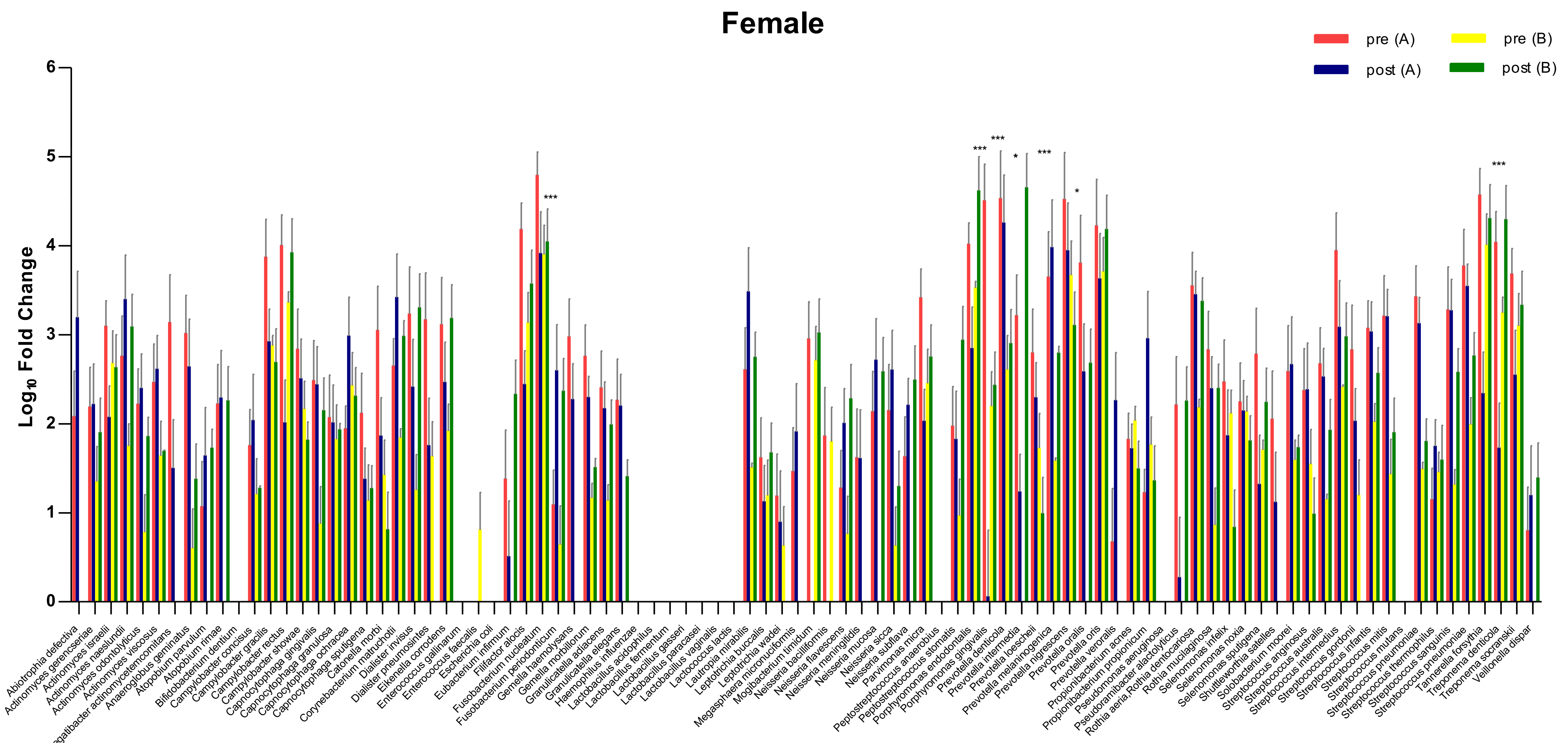
Female
pre (A)
post (A)
pre (B)
post (B)
Log10 Fold Change
0
1
2
3
4
5
6
***
*
Abiotrophia defectiva
Actinomyces gerencseriae
Actinomyces israelii
Actinomyces naeslundii
Actinomyces odontolyticus
Actinomyces viscosus
Aggregatibacter actinomycetemcomitans
Anaeroglobus geminatus
Atopobium parvulum
Atopobium rimae
Bifidobacterium dentium
Campylobacter concisus
Campylobacter gracilis
Campylobacter rectus
Campylobacter showae
Capnocytophaga gingivalis
Capnocytophaga granulosa
Capnocytophaga ochracea
Capnocytophaga sputigena
Catonella morbi
Corynebacterium matruchotii
Dialister invisus
Dialister pneumosintes
Eikenella corrodens
Enterococcus gallinarum
Enterococcus faecalis
Escherichia coli
Eubacterium infirmum
Filifactor alocis
Fusobacterium nucleatum
Fusobacterium periodonticum
Gemella haemolysans
Gemella morbillorum
Granulicatella adiacens
Granulicatella elegans
Haemophilus influenzae
Lactobacillus acidophilus
Lactobacillus fermentum
Lactobacillus gasseri
Lactobacillus paracasei
Lactobacillus vaginalis
Lactococcus lactis
Lautropia mirabilis
Leptotrichia buccalis
Leptotrichia wadei
Megasphaera micronuciformis
Mogibacterium timidum
Neisseria bacilliformis
Neisseria flavescens
Neisseria meningitidis
Neisseria mucosa
Neisseria sicca
Neisseria subflava
Parvimonas micra
Peptostreptococcus anaerobius
Peptostreptococcus stomatis
Porphyromonas endodontalis
Porphyromonas gingivalis
Prevotella denticola
Prevotella intermedia
Prevotella loescheii
Prevotella melaninogenica
Prevotella nigrescens
Prevotella oralis
Prevotella veroralis
Propionibacterium acnes
Propionibacterium propionicum
Pseudomonas aeruginosa
Pseudoramibacter alactolyticus
Rothia aeria
Rothia dentocariosa
Rothia mucilaginosa
Selenomonas infelix
Selenomonas noxia
Selenomonas sputigena
Shuttleworthia satelles
Solobacterium moorei
Streptococcus anginosus
Streptococcus australis
Streptococcus intermedius
Streptococcus gordonii
Streptococcus infantis
Streptococcus mitis
Streptococcus mutans
Streptococcus pneumoniae
Streptococcus thermophilus
Streptococcus sanguinis
Streptococcus pneumoniae
Tannerella forsythia
Treponema denticola
Treponema socranskii
Veillonella dispar

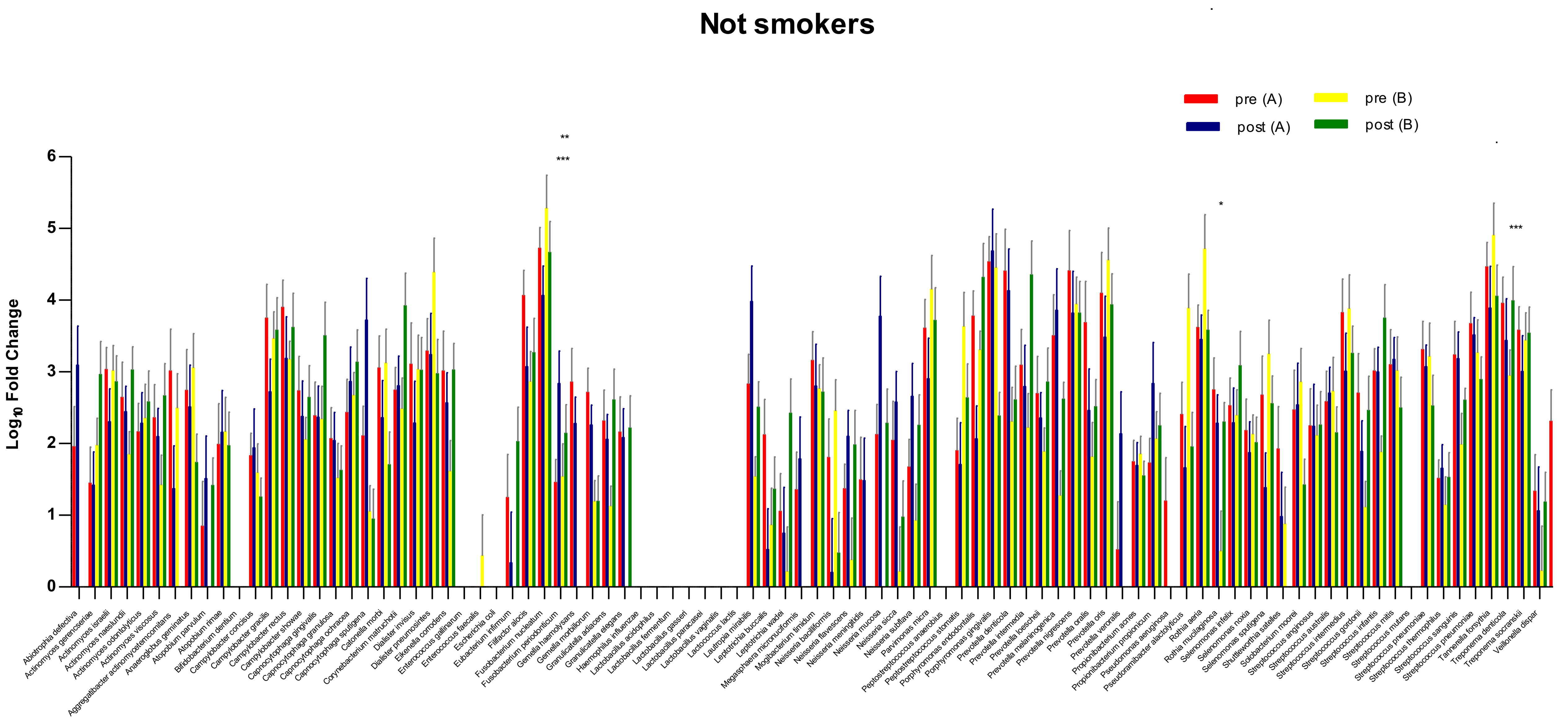
Not smokers
pre (A)
post (A)
pre (B)
post (B)
Log10 Fold Change
0
1
2
3
4
5
6
**
***
*
***
Abiotrophia defectiva
Actinomyces gerencseriae
Actinomyces israelii
Actinomyces naeslundii
Actinomyces odontolyticus
Actinomyces viscosus
Aggregatibacter actinomycetemcomitans
Anaeroglobus geminatus
Atopobium parvulum
Atopobium rimae
Bifidobacterium dentium
Campylobacter concisus
Campylobacter gracilis
Campylobacter rectus
Campylobacter showae
Capnocytophaga gingivalis
Capnocytophaga granulosa
Capnocytophaga ochracea
Capnocytophaga sputigena
Catonella morbi
Corynebacterium matruchotii
Dialister invisus
Dialister pneumosintes
Eikenella corrodens
Enterococcus gallinarum
Enterococcus faecalis
Escherichia coli
Eubacterium infirmum
Filifactor alocis
Fusobacterium nucleatum
Fusobacterium periodonticum
Gemella haemolysans
Gemella morbillorum
Granulicatella adiacens
Granulicatella elegans
Haemophilus influenzae
Lactobacillus acidophilus
Lactobacillus fermentum
Lactobacillus gasseri
Lactobacillus paracasei
Lactobacillus vaginalis
Lactococcus lactis
Lautropia mirabilis
Leptotrichia buccalis
Leptotrichia wadei
Megasphaera micronuciformis
Mogibacterium timidum
Neisseria bacilliformis
Neisseria flavescens
Neisseria meningitidis
Neisseria mucosa
Neisseria sicca
Neisseria subflava
Parvimonas micra
Peptostreptococcus anaerobius
Peptostreptococcus stomatis
Porphyromonas endodontalis
Porphyromonas gingivalis
Prevotella denticola
Prevotella intermedia
Prevotella loescheii
Prevotella melaninogenica
Prevotella nigrescens
Prevotella oralis
Prevotella oris
Prevotella veroralis
Propionibacterium acnes
Propionibacterium propionicum
Pseudomonas aeruginosa
Pseudoramibacter alactolyticus
Rothia aeria
Rothia mucilaginosa
Selenomonas infelix
Selenomonas noxia
Selenomonas sputigena
Shuttleworthia satelles
Solobacterium moorei
Streptococcus anginosus
Streptococcus australis
Streptococcus intermedius
Streptococcus gordonii
Streptococcus infantis
Streptococcus mitis
Streptococcus mutans
Streptococcus pneumoniae
Streptococcus thermophilus
Streptococcus sanguinis
Streptococcus pneumoniae
Tannerella forsythia
Treponema denticola
Treponema socranskii
Veillonella dispar

Smoking

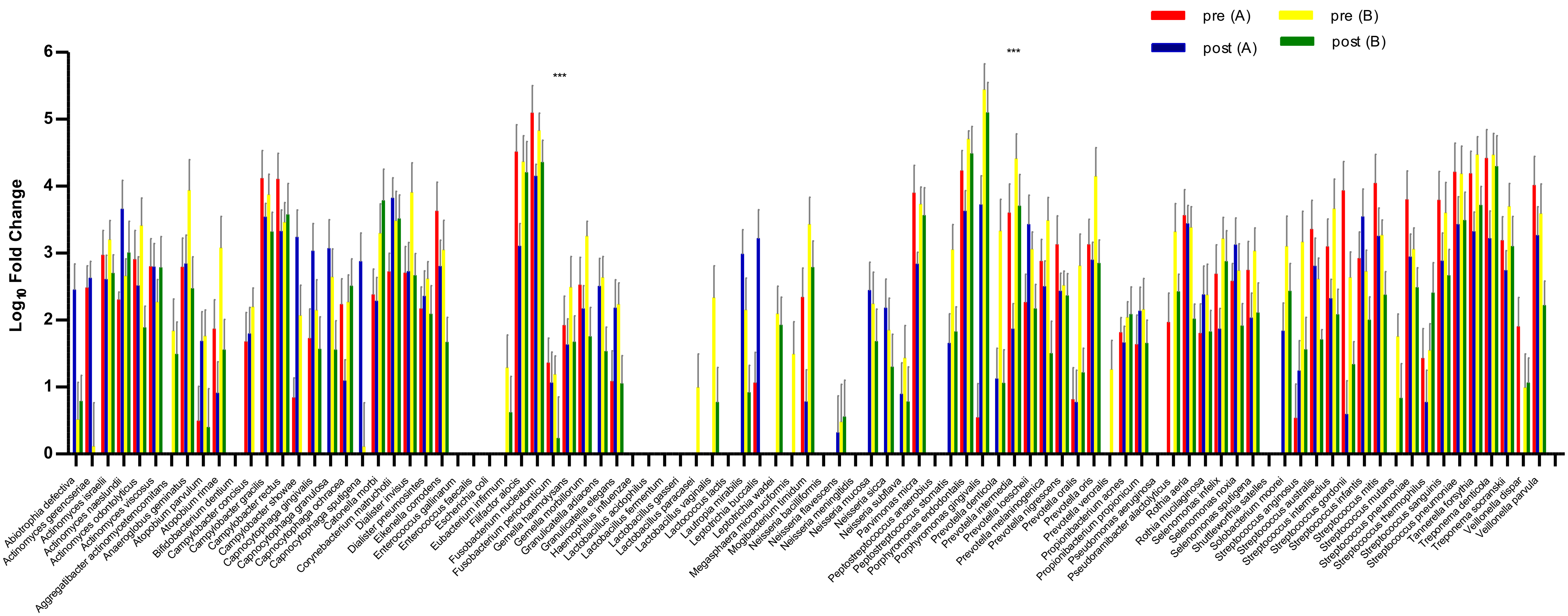
pre (A)
post (A)
pre (B)
post (B)
Log10 Fold Change
0
1
2
3
4
5
6
***
***
Abiotrophia defectiva
Actinomyces gerencseriae
Actinomyces israelii
Actinomyces naeslundii
Actinomyces odontolyticus
Actinomyces viscosus
Aggregatibacter actinomycetemcomitans
Anaeroglobus geminatus
Atopobium parvulum
Atopobium rimae
Bifidobacterium dentium
Campylobacter concisus
Campylobacter gracilis
Campylobacter rectus
Campylobacter showae
Capnocytophaga gingivalis
Capnocytophaga granulosa
Capnocytophaga ochracea
Capnocytophaga sputigena
Catonella morbi
Corynebacterium matruchotii
Dialister invisus
Dialister pneumosintes
Eikenella corrodens
Enterococcus gallinarum
Enterococcus faecalis
Escherichia coli
Eubacterium infirmum
Filifactor alocis
Fusobacterium nucleatum
Fusobacterium periodonticum
Gemella haemolysans
Gemella morbillorum
Granulicatella adiacens
Granulicatella elegans
Haemophilus influenzae
Lactobacillus acidophilus
Lactobacillus fermentum
Lactobacillus gasseri
Lactobacillus paracasei
Lactobacillus vaginalis
Lactococcus lactis
Lautropia mirabilis
Leptotrichia buccalis
Leptotrichia wadei
Megasphaera micronuciformis
Mogibacterium timidum
Neisseria bacilliformis
Neisseria flavescens
Neisseria meningitidis
Neisseria mucosa
Neisseria sicca
Neisseria subflava
Parvimonas micra
Peptostreptococcus anaerobius
Peptostreptococcus stomatis
Porphyromonas endodontalis
Porphyromonas gingivalis
Prevotella denticola
Prevotella intermedia
Prevotella loescheii
Prevotella melaninogenica
Prevotella nigrescens
Prevotella oralis
Prevotella oris
Prevotella veroralis
Propionibacterium acnes
Propionibacterium propionicum
Pseudomonas aeruginosa
Pseudoramibacter alactolyticus
Rothia aeria
Rothia mucilaginosa
Selenomonas infelix
Selenomonas noxia
Selenomonas sputigena
Shuttleworthia satelles
Solobacterium moorei
Streptococcus anginosus
Streptococcus australis
Streptococcus intermedius
Streptococcus gordonii
Streptococcus infantis
Streptococcus mitis
Streptococcus mutans
Streptococcus pneumoniae
Streptococcus thermophilus
Streptococcus sanguinis
Streptococcus pneumoniae
Tannerella forsythia
Treponema denticola
Treponema socranskii
Veillonella dispar
Veillonella parvula

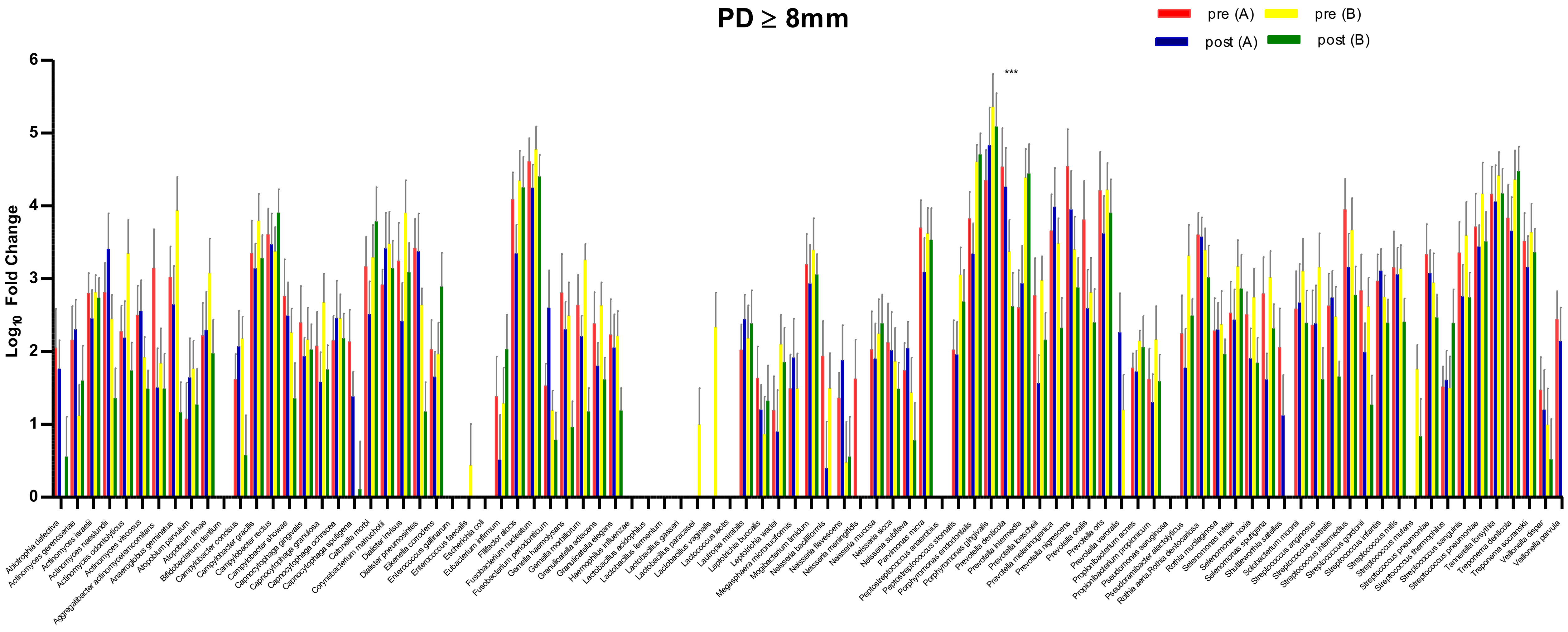
PD ≥ 8mm
pre (A)
pre (B)
post (A)
post (B)
Log10 Fold Change
***
0
1
2
3
4
5
6
Abiotrophia defectiva
Actinomyces gerencseriae
Actinomyces israelii
Actinomyces naeslundii
Actinomyces odontolyticus
Actinomyces viscosus
Aggregatibacter actinomycetemcomitans
Anaeroglobus geminatus
Atopobium parvulum
Atopobium rimae
Bifidobacterium dentium
Campylobacter concisus
Campylobacter gracilis
Campylobacter rectus
Campylobacter showae
Capnocytophaga gingivalis
Capnocytophaga granulosa
Capnocytophaga ochracea
Capnocytophaga sputigena
Catonella morbi
Corynebacterium matruchotii
Dialister invisus
Dialister pneumosintes
Eikenella corrodens
Enterococcus gallinarum
Enterococcus faecalis
Escherichia coli
Eubacterium infirmum
Filifactor alocis
Fusobacterium nucleatum
Fusobacterium periodonticum
Gemella haemolysans
Gemella morbillorum
Granulicatella adiacens
Granulicatella elegans
Haemophilus influenzae
Lactobacillus acidophilus
Lactobacillus fermentum
Lactobacillus gasseri
Lactobacillus paracasei
Lactobacillus vaginalis
Lactococcus lactis
Lautropia mirabilis
Leptotrichia buccalis
Leptotrichia wadei
Megasphaera micronuciformis
Mogibacterium timidum
Neisseria bacilliformis
Neisseria flavescens
Neisseria meningitidis
Neisseria mucosa
Neisseria sicca
Neisseria subflava
Parvimonas micra
Peptostreptococcus anaerobius
Peptostreptococcus stomatis
Porphyromonas endodontalis
Porphyromonas gingivalis
Prevotella denticola
Prevotella intermedia
Prevotella loescheii
Prevotella melaninogenica
Prevotella nigrescens
Prevotella oralis
Prevotella oris
Prevotella veroralis
Propionibacterium acnes
Propionibacterium propionicum
Pseudomonas aeruginosa
Pseudoramibacter alactolyticus
Rothia aeria,Rothia dentocariosa
Rothia mucilaginosa
Selenomonas infelix
Selenomonas noxia
Selenomonas sputigena
Shuttleworthia satelles
Solobacterium moorei
Streptococcus anginosus
Streptococcus australis
Streptococcus intermedius
Streptococcus gordonii
Streptococcus infantis
Streptococcus mitis
Streptococcus mutans
Streptococcus pneumoniae
Streptococcus thermophilus
Streptococcus sanguinis
Streptococcus pneumoniae
Tannerella forsythia
Treponema denticola
Treponema socranskii
Veillonella dispar
Veillonella parvula

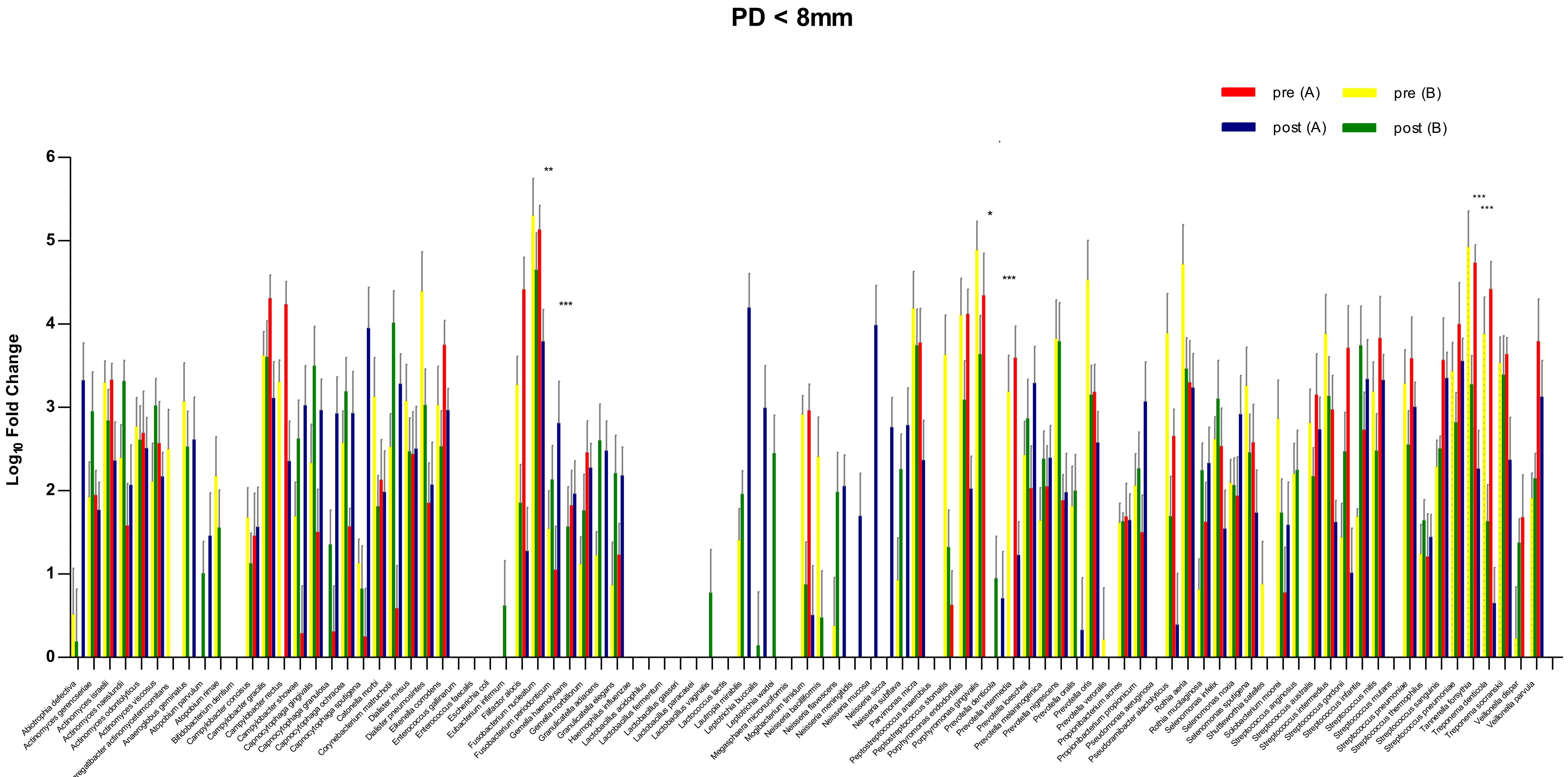
PD < 8mm
pre (A)
pre (B)
post (A)
post (B)
Log10 Fold Change
0
1
2
3
4
5
6
**
***
*
***
***
***
Abiotrophia defectiva
Actinomyces gerencseriae
Actinomyces israelii
Actinomyces naeslundii
Actinomyces odontolyticus
Actinomyces viscosus
Aggregatibacter actinomycetemcomitans
Anaeroglobus geminatus
Atopobium parvulum
Atopobium rimae
Bifidobacterium dentium
Campylobacter concisus
Campylobacter gracilis
Campylobacter rectus
Campylobacter showae
Capnocytophaga gingivalis
Capnocytophaga granulosa
Capnocytophaga ochracea
Capnocytophaga sputigena
Catonella morbi
Corynebacterium matruchotii
Dialister invisus
Dialister pneumosintes
Eikenella corrodens
Enterococcus gallinarum
Enterococcus faecalis
Escherichia coli
Eubacterium infirmum
Filifactor alocis
Fusobacterium nucleatum
Fusobacterium periodonticum
Gemella haemolysans
Gemella morbillorum
Granulicatella adiacens
Granulicatella elegans
Haemophilus influenzae
Lactobacillus acidophilus
Lactobacillus fermentum
Lactobacillus gasseri
Lactobacillus paracasei
Lactobacillus vaginalis
Lactococcus lactis
Lautropia mirabilis
Leptotrichia buccalis
Leptotrichia wadei
Megasphaera micronuciformis
Mogibacterium timidum
Neisseria bacilliformis
Neisseria flavescens
Neisseria meningitidis
Neisseria mucosa
Neisseria sicca
Neisseria subflava
Parvimonas micra
Peptostreptococcus anaerobius
Peptostreptococcus stomatis
Porphyromonas endodontalis
Porphyromonas gingivalis
Prevotella denticola
Prevotella intermedia
Prevotella loescheii
Prevotella melaninogenica
Prevotella nigrescens
Prevotella oralis
Prevotella oris
Prevotella veroralis
Propionibacterium acnes
Propionibacterium propionicum
Pseudomonas aeruginosa
Pseudoramibacter alactolyticus
Rothia aeria
Rothia mucilaginosa
Selenomonas infelix
Selenomonas noxia
Selenomonas sputigena
Shuttleworthia satelles
Solobacterium moorei
Streptococcus anginosus
Streptococcus australis
Streptococcus intermedius
Streptococcus gordonii
Streptococcus infantis
Streptococcus mitis
Streptococcus mutans
Streptococcus pneumoniae
Streptococcus thermophilus
Streptococcus sanguinis
Streptococcus pneumoniae
Tannerella forsythia
Treponema denticola
Treponema socranskii
Veillonella dispar
Veillonella parvula

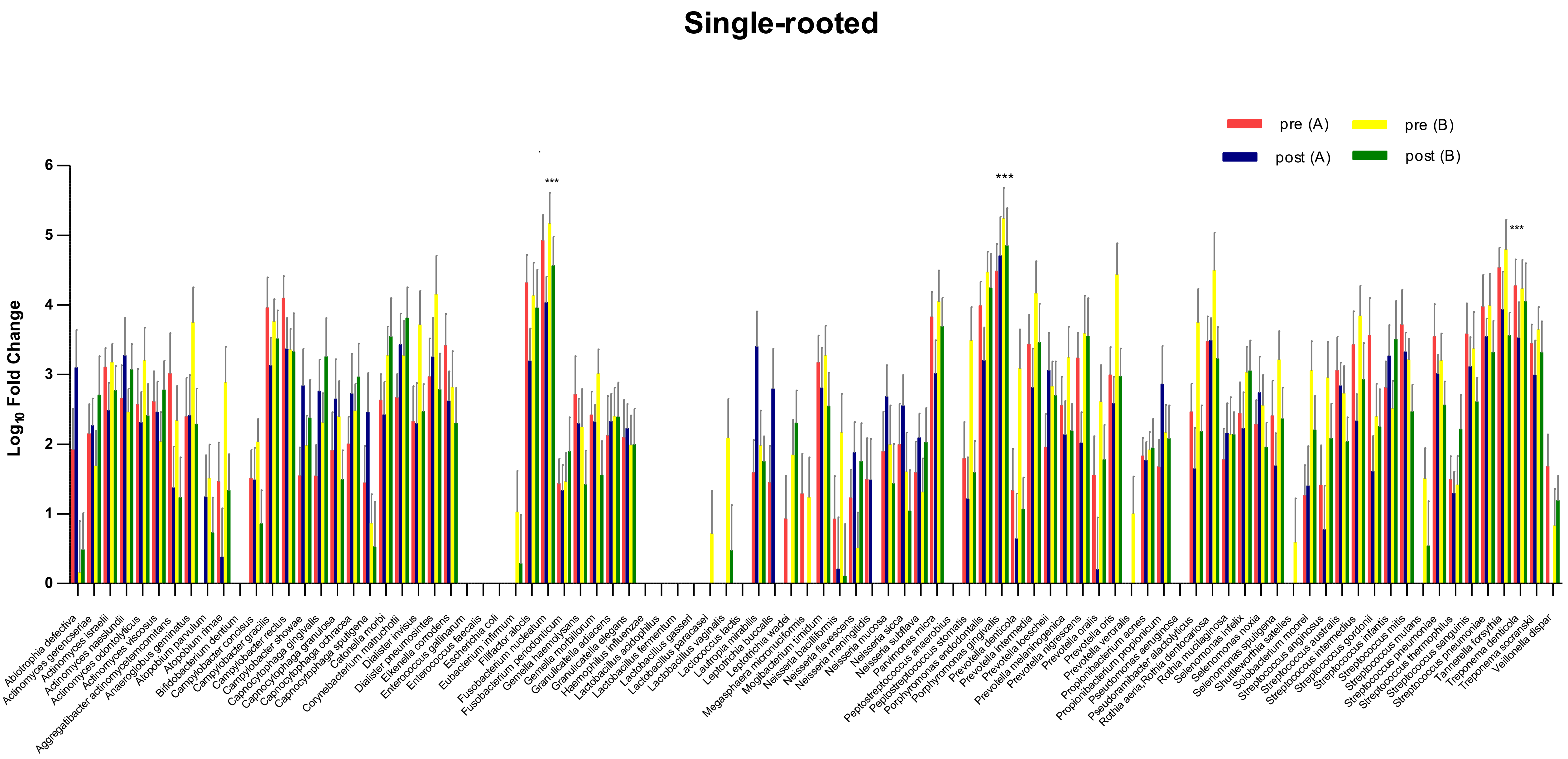

Single-rooted
pre (A)
pre (B)
post (A)
post (B)
Log10 Fold Change
***
***
***
Abiotrophia defectiva
Actinomyces gerencseriae
Actinomyces israelii
Actinomyces naeslundii
Actinomyces odontolyticus
Actinomyces viscosus
Aggregatibacter actinomycetemcomitans
Anaeroglobus geminatus
Atopobium parvulum
Atopobium rimae
Bifidobacterium dentium
Campylobacter concisus
Campylobacter gracilis
Campylobacter rectus
Campylobacter showae
Capnocytophaga gingivalis
Capnocytophaga granulosa
Capnocytophaga ochracea
Capnocytophaga sputigena
Catonella morbi
Corynebacterium matruchotii
Dialister invisus
Dialister pneumosintes
Eikenella corrodens
Enterococcus gallinarum
Enterococcus faecalis
Escherichia coli
Eubacterium infirmum
Filifactor alocis
Fusobacterium nucleatum
Fusobacterium periodonticum
Gemella haemolysans
Gemella morbillorum
Granulicatella adiacens
Granulicatella elegans
Haemophilus influenzae
Lactobacillus acidophilus
Lactobacillus fermentum
Lactobacillus gasseri
Lactobacillus paracasei
Lactobacillus vaginalis
Lactococcus lactis
Lautropia mirabilis
Leptotrichia buccalis
Leptotrichia wadei
Megasphaera micronuciformis
Mogibacterium timidum
Neisseria bacilliformis
Neisseria flavescens
Neisseria meningitidis
Neisseria mucosa
Neisseria sicca
Neisseria subflava
Parvimonas micra
Peptostreptococcus anaerobius
Peptostreptococcus stomatis
Porphyromonas endodontalis
Porphyromonas gingivalis
Prevotella denticola
Prevotella intermedia
Prevotella loescheii
Prevotella melaninogenica
Prevotella nigrescens
Prevotella oralis
Prevotella oris
Prevotella veroralis
Propionibacterium acnes
Propionibacterium propionicum
Pseudomonas aeruginosa
Pseudoramibacter alactolyticus
Rothia aeria
Rothia dentocariosa
Rothia mucilaginosa
Selenomonas infelix
Selenomonas noxia
Selenomonas sputigena
Shuttleworthia satelles
Solobacterium moorei
Streptococcus anginosus
Streptococcus australis
Streptococcus intermedius
Streptococcus gordonii
Streptococcus infantis
Streptococcus mitis
Streptococcus mutans
Streptococcus pneumoniae
Streptococcus thermophilus
Streptococcus sanguinis
Streptococcus pneumoniae
Tannerella forsythia
Treponema denticola
Treponema socranskii
Veillonella dispar

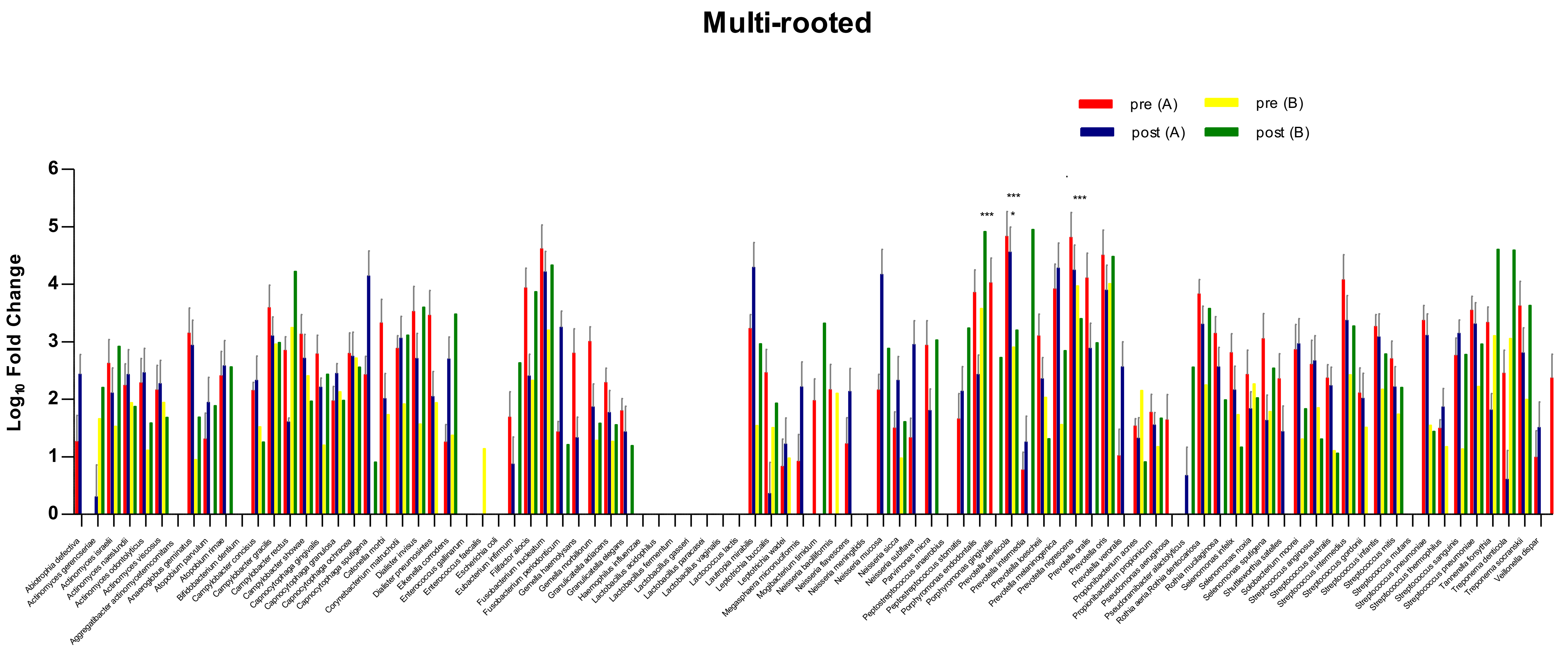
Multi-rooted
pre (A)
pre (B)
post (A)
post (B)
Log10 Fold Change
0
1
2
3
4
5
6
***
***
*
***
Abiotrophia defectiva
Actinomyces gerencseriae
Actinomyces israelii
Actinomyces naeslundii
Actinomyces odontolyticus
Actinomyces viscosus
Aggregatibacter actinomycetemcomitans
Anaeroglobus geminatus
Atopobium parvulum
Atopobium rimae
Bifidobacterium dentium
Campylobacter concisus
Campylobacter gracilis
Campylobacter rectus
Campylobacter showae
Capnocytophaga gingivalis
Capnocytophaga granulosa
Capnocytophaga ochracea
Capnocytophaga sputigena
Catonella morbi
Corynebacterium matruchotii
Dialister invisus
Dialister pneumosintes
Eikenella corrodens
Enterococcus gallinarum
Enterococcus faecalis
Escherichia coli
Eubacterium infirmum
Filifactor alocis
Fusobacterium nucleatum
Fusobacterium periodonticum
Gemella haemolysans
Gemella morbillorum
Granulicatella adiacens
Granulicatella elegans
Haemophilus influenzae
Lactobacillus acidophilus
Lactobacillus fermentum
Lactobacillus gasseri
Lactobacillus paracasei
Lactobacillus vaginalis
Lactococcus lactis
Lautropia mirabilis
Leptotrichia buccalis
Leptotrichia wadei
Megasphaera micronuciformis
Mogibacterium timidum
Neisseria bacilliformis
Neisseria flavescens
Neisseria meningitidis
Neisseria mucosa
Neisseria sicca
Neisseria subflava
Parvimonas micra
Peptostreptococcus anaerobius
Peptostreptococcus stomatis
Porphyromonas endodontalis
Porphyromonas gingivalis
Prevotella denticola
Prevotella intermedia
Prevotella loescheii
Prevotella melaninogenica
Prevotella nigrescens
Prevotella oralis
Prevotella oris
Prevotella veroralis
Propionibacterium acnes
Propionibacterium propionicum
Pseudomonas aeruginosa
Pseudoramibacter alactolyticus
Rothia aeria;Rothia dentocariosa
Rothia mucilaginosa
Selenomonas infelix
Selenomonas noxia
Selenomonas sputigena
Shuttleworthia satelles
Solobacterium moorei
Streptococcus anginosus
Streptococcus australis
Streptococcus intermedius
Streptococcus gordonii
Streptococcus infantis
Streptococcus mitis
Streptococcus mutans
Streptococcus pneumoniae
Streptococcus thermophilus
Streptococcus sanguinis
Streptococcus pneumoniae
Tannerella forsythia
Treponema denticola
Treponema socranskii
Veillonella dispar

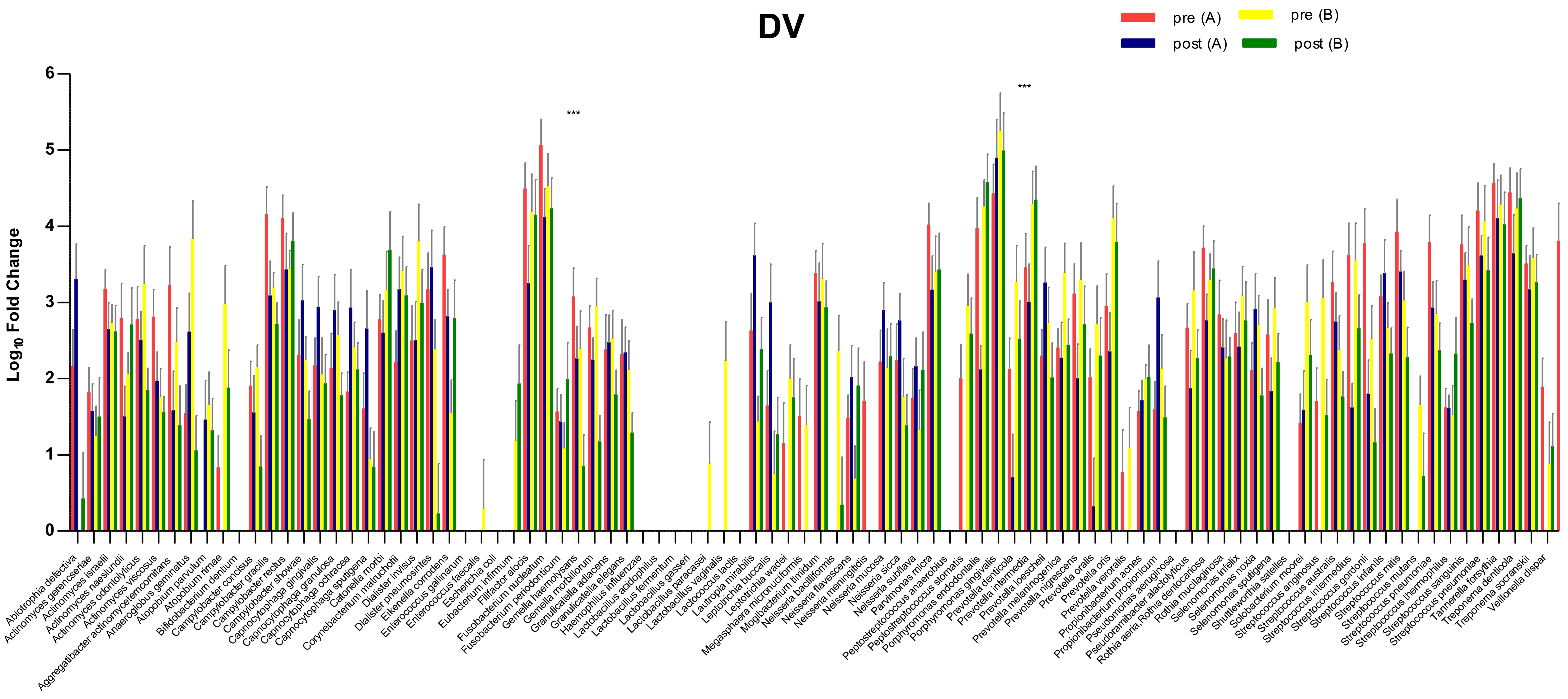
DV
pre (A)
pre (B)
post (A)
post (B)
Log10 Fold Change
0
1
2
3
4
5
6
***
***
Abiotrophia defectiva
Actinomyces gerencseriae
Actinomyces israelii
Actinomyces naeslundii
Actinomyces odontolyticus
Actinomyces viscosus
Aggregatibacter actinomycetemcomitans
Anaeroglobus geminatus
Atopobium parvulum
Atopobium rimae
Bifidobacterium dentium
Campylobacter concisus
Campylobacter gracilis
Campylobacter rectus
Campylobacter showae
Capnocytophaga gingivalis
Capnocytophaga granulosa
Capnocytophaga ochracea
Capnocytophaga sputigena
Catonella morbi
Corynebacterium matruchotii
Dialister invisus
Dialister pneumosintes
Eikenella corrodens
Enterococcus gallinarum
Enterococcus faecalis
Escherichia coli
Eubacterium infirmum
Filifactor alocis
Fusobacterium nucleatum
Fusobacterium periodonticum
Gemella haemolysans
Gemella morbillorum
Granulicatella adiacens
Granulicatella elegans
Haemophilus influenzae
Lactobacillus acidophilus
Lactobacillus fermentum
Lactobacillus gasseri
Lactobacillus paracasei
Lactobacillus vaginalis
Lactococcus lactis
Lautropia mirabilis
Leptotrichia buccalis
Leptotrichia wadei
Megasphaera micronuciformis
Mogibacterium timidum
Neisseria bacilliformis
Neisseria flavescens
Neisseria meningitidis
Neisseria mucosa
Neisseria sicca
Neisseria subflava
Parvimonas micra
Peptostreptococcus anaerobius
Peptostreptococcus stomatis
Porphyromonas endodontalis
Porphyromonas gingivalis
Prevotella denticola
Prevotella intermedia
Prevotella loescheii
Prevotella melaninogenica
Prevotella nigrescens
Prevotella oralis
Prevotella oris
Prevotella veroralis
Propionibacterium acnes
Propionibacterium propionicum
Pseudomonas aeruginosa
Pseudoramibacter alactolyticus
Rothia aeria,Rothia dentocariosa
Rothia mucilaginosa
Selenomonas infelix
Selenomonas noxia
Selenomonas sputigena
Shuttleworthia satelles
Solobacterium moorei
Streptococcus anginosus
Streptococcus australis
Streptococcus intermedius
Streptococcus gordonii
Streptococcus infantis
Streptococcus mitis
Streptococcus mutans
Streptococcus pneumoniae
Streptococcus thermophilus
Streptococcus sanguinis
Streptococcus pneumoniae
Tannerella forsythia
Treponema denticola
Treponema socranskii
Veillonella dispar

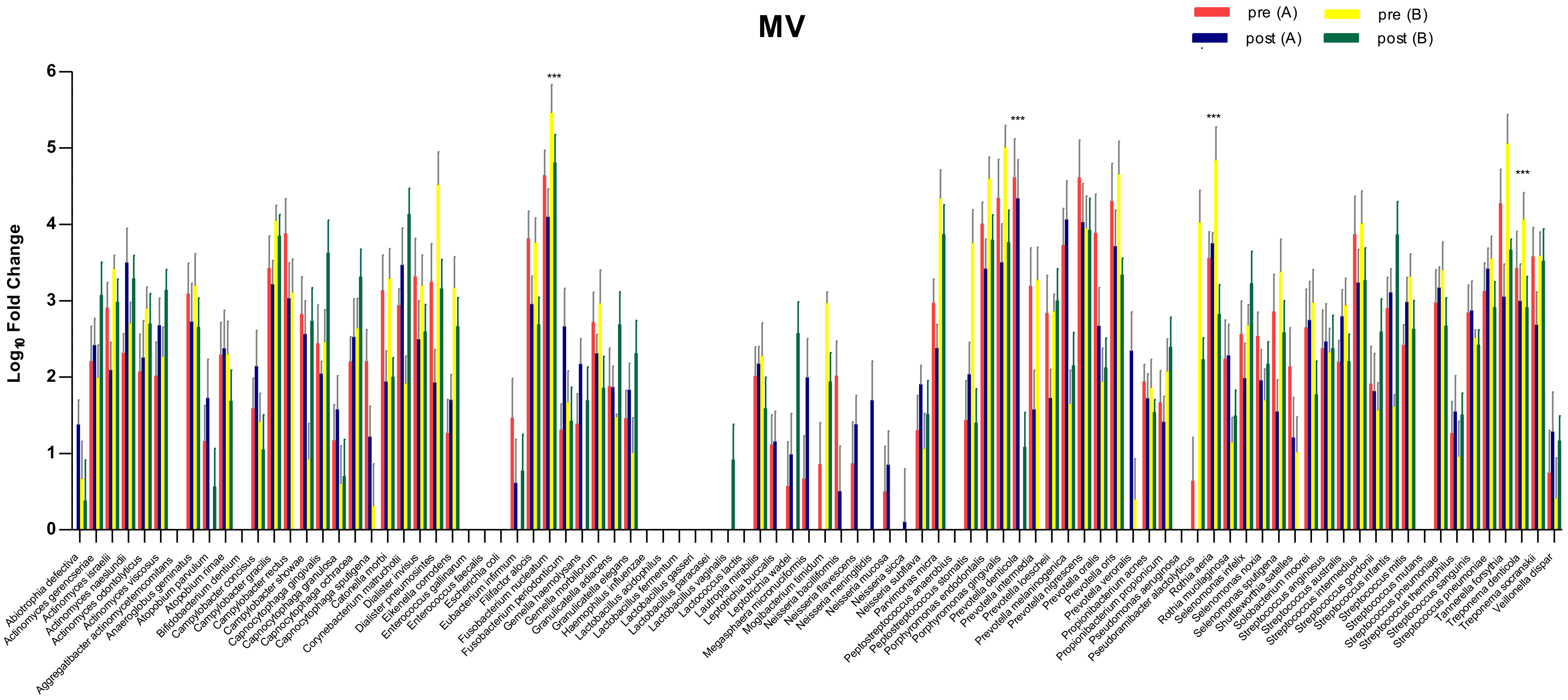
MV
pre (A)
pre (B)
post (A)
post (B)
Log_{10} Fold Change
0
1
2
3
4
5
6
***
***
***
***
Abiotrophia defectiva
Actinomyces gerencseriae
Actinomyces israelii
Actinomyces naeslundii
Actinomyces odontolyticus
Actinomyces viscosus
Aggregatibacter actinomycetemcomitans
Anaeroglobus geminatus
Atopobium parvulum
Atopobium rimae
Bifidobacterium dentium
Campylobacter concisus
Campylobacter gracilis
Campylobacter rectus
Campylobacter showae
Capnocytophaga gingivalis
Capnocytophaga granulosa
Capnocytophaga ochracea
Capnocytophaga sputigena
Catonella morbi
Corynebacterium matruchotii
Dialister invisus
Dialister pneumosintes
Eikenella corrodens
Enterococcus gallinarum
Enterococcus faecalis
Escherichia coli
Eubacterium infirmum
Filifactor alocis
Fusobacterium nucleatum
Fusobacterium periodonticum
Gemella haemolysans
Gemella morbillorum
Granulicatella adiacens
Granulicatella elegans
Haemophilus influenzae
Lactobacillus acidophilus
Lactobacillus fermentum
Lactobacillus gasseri
Lactobacillus paracasei
Lactobacillus vaginalis
Lactococcus lactis
Lautropia mirabilis
Leptotrichia buccalis
Leptotrichia wadei
Megasphaera micronuciformis
Mogibacterium timidum
Neisseria bacilliformis
Neisseria flavescens
Neisseria meningitidis
Neisseria mucosa
Neisseria sicca
Neisseria subflava
Parvimonas micra
Peptostreptococcus anaerobius
Peptostreptococcus stomatis
Porphyromonas endodontalis
Porphyromonas gingivalis
Prevotella denticola
Prevotella intermedia
Prevotella loescheii
Prevotella melaninogenica
Prevotella nigrescens
Prevotella oralis
Prevotella oris
Prevotella veroralis
Propionibacterium acnes
Propionibacterium propionicum
Pseudomonas aeruginosa
Pseudoramibacter alactolyticus
Rothia aeria
Rothia mucilaginosa
Selenomonas infelix
Selenomonas noxia
Selenomonas sputigena
Shuttleworthia satelles
Solobacterium moorei
Streptococcus anginosus
Streptococcus australis
Streptococcus intermedius
Streptococcus gordonii
Streptococcus infantis
Streptococcus mitis
Streptococcus mutans
Streptococcus pneumoniae
Streptococcus thermophilus
Streptococcus sanguinis
Streptococcus pneumoniae
Tannerella forsythia
Treponema denticola
Treponema socranskii
Veillonella dispar
